# Screening for peptides targeted to IL-7Rα for molecular imaging of rheumatoid arthritis synovium

**DOI:** 10.1186/s13075-016-1133-8

**Published:** 2016-10-12

**Authors:** Carmen Burtea, Sophie Laurent, Tuba Sanli, Deborah Fanfone, Aude Devalckeneer, Sébastien Sauvage, Marie-Claire Beckers, Sandrine Rorive, Isabelle Salmon, Luce Vander Elst, Bernard R. Lauwerys, Robert N. Muller

**Affiliations:** 1 Department of General, Organic and Biomedical Chemistry, NMR and Molecular Imaging Laboratory, University of Mons, Avenue Maistriau 19, Mendeleev Building, Mons, B-7000 Belgium; 2 Department of Pathology, Erasme Hospital, Université Libre de Bruxelles, Route de Lennik 808, Brussels, 1070 Belgium; 3 DIAPath, Center for Microscopy and Molecular Imaging, 8, rue Adrienne Bolland, Gosselies, 6041 Belgium; 4 Eurogentec S.A., Liège Science Park, Rue du Bois Saint-Jean 5, Seraing, B-4102 Belgium; 5 Center for Microscopy and Molecular Imaging, 8, rue Adrienne Bolland, Gosselies, 6041 Belgium; 6 Pôle de pathologies rhumatismales inflammatoires et systémiques, Institut de Recherche Expérimentale et Clinique, Université Catholique de Louvain, Avenue Mounier 53, Brussels, 1200 Belgium; 7 Present address: Molecular Pathology Laboratory, ONCODNA – The Cancer Theranostic Company, 25 Av. Georges Lemaître, Gosselies, 6041 Belgium; 8 Present address: ASIT Biotech s.a, 3 Rue des Chasseurs Ardennais, Angleur, 4031 Belgium

**Keywords:** Rheumatoid arthritis, IL-7 receptor, Ultra-small superparamagnetic particles of iron oxide, MRI, Molecular imaging, Peptides, Phage display

## Abstract

**Background:**

Interleukin-7 receptor alpha (IL-7Rα) represents a biomarker with potential applications in rheumatoid arthritis (RA) diagnosis and therapy. We have therefore searched by phage display potential IL-7Rα specific peptides with the primary goal being to develop in vivo molecular imaging tools.

**Methods:**

IL-7Rα-targeted peptides were searched within a disulfide-constrained combinatorial phage displayed library of random linear heptapeptides. The apparent dissociation constant (K_d_) and half maximal inhibition constant (IC_50_) were estimated for phage clones and synthesized peptides by ELISA. We used 5-Aza-2’-deoxycytidine (ADC)-stimulated Jurkat cells and human synovial tissue from patients with RA for in vitro characterization of peptides. For molecular imaging studies performed by magnetic resonance imaging (MRI), experimental arthritis was induced in DBA/1 male mice by immunization with an emulsion of complete Freund’s adjuvant and type II collagen from chicken sternal cartilage.

**Results:**

After several steps of phage display and peptide screening, two IL-7Rα-specific heptapeptides (P258 and P725) were selected from the initial library, based on their affinity for the target (extracellular domain of IL-7Rα, which contains a fibronectin type III repeat-like sequence). P258 (a linear peptide obtained by removing the Cys-constraint) had the lowest affinity for fibronectin itself and was therefore proposed for molecular imaging. After grafting to ultra-small superparamagnetic particles of iron oxide (USPIO), P258 produced a strong negative contrast on MRI in mice with collagen-induced arthritis (CIA), even at 2 hours post injection. The co-localization of USPIO-P258 with IL-7Rα-expressing cells in the synovial tissue from CIA mice and its ability to discriminate the level of IL-7R expression and the disease severity confirmed its efficacy as an in vivo IL-7Rα imaging agent. Interestingly, the cyclic peptide (P725), which was less adequate for molecular imaging because of higher affinity for fibronectin, had a strong ability to compete with IL-7 for the IL-7Rα binding sites, making it a potential candidate for blocking applications. Accordingly, P725 prevented the signal transducer and activator of transcription 5 (STAT5) activation induced by IL-7 in ADC-stimulated Jurkat cells.

**Conclusions:**

The two peptides identified in this work demonstrate that IL-7Rα targeting in RA presents potential applications for in vivo molecular imaging and putative blocking purposes.

**Electronic supplementary material:**

The online version of this article (doi:10.1186/s13075-016-1133-8) contains supplementary material, which is available to authorized users.

## Background

Rheumatoid arthritis (RA) is a chronic and debilitating autoimmune disease that causes joint damage, decreased quality of life and cardiovascular complications, among other comorbidities. With 5–50 per 100,000 new cases annually, RA occurs in 0.5–1 % of adults in industrialized countries, being more frequent in women and elderly people [1].

Interleukin-7 (IL-7) is an anti-apoptotic cytokine, essential for T cell proliferation, development and homeostasis. It is also involved in B cell development. The IL-7 receptor (IL-7R) comprises two heterodimeric subunits, IL-7Rα and the common γ-chain (γc) respectively. IL-7Rα is composed of a 195 amino acid intracellular domain, a 25 amino acid transmembrane domain and an extracellular region comprising 219 amino acids. Four invariant cysteine residues located at the N-terminus of the extracellular domain are involved in intrachain disulfide bond formation. A Trp-Ser-X-Trp-Ser (WSXWS) motif is located close to the transmembrane domain of the extracellular region of IL-7Rα, which also contains a fibronectin (FN) type III-like domain. The intracellular domain of IL-7Rα is responsible of signal transduction via the recruitment of signal transducing molecules, such as the Janus kinase 1 (Jak1), the signal transducer and activator of transcription 5 (STAT5) and the src family of tyrosine kinases, and is involved in the IL-7-dependent activation of phosphatidylinositol 3-kinase (PI3K) [2, 3]. IL-7 binding to its receptor triggers several signaling cascades, i.e., Jak/STAT, PI3K, Ras and mitogen-activated protein kinase (MAPK)/extracellular signal-related kinase (ERK), being essential for lymphocyte survival, homeostasis and differentiation [2–4].

IL-7 and IL-7R are over-expressed in the RA synovium. IL-7 plays a crucial role in T cell activation and osteoclastogenesis by upregulating T cell-derived cytokines, including the receptor activator of nuclear factor-κB ligand (RANKL) [5]. In RA synovitis, not only T cells, but also synovial macrophages and fibroblasts over-express IL-7Rα, thereby making IL-7Rα the transcript most differentially expressed between RA and other inflammatory joint conditions such as osteoarthritis, systemic lupus erythematosus, psoriatic arthritis and gout [6–8]. Synovial fibroblasts also produce a high quantity of soluble IL-7Rα subsequent to their stimulation by cytokines such as tumor necrosis factor-α (TNFα), IL-1 and IL-17 [9–11]. According to several observations, soluble IL-7Rα stabilizes IL-7 and amplifies its T cell stimulatory effects [12]. The increased expression and high serum concentrations of soluble IL-7Rα is associated with poor response to anti-TNFα therapy in patients with RA [13, 14]. The pathogenic role of the IL-7/IL-7R axis in RA is further illustrated in a mouse model of the disease (collagen-induced arthritis, CIA), in which both IL-7 or IL-7R blockade using monoclonal antibodies results in significant improvements in disease activity [15–17]. Of note, recent observations also highlight the potential involvement of IL-7/IL-7R in other autoimmune disorders, such as systemic lupus erythematosus and Sjögren’s syndrome [18, 19].

As it is currently an incurable disease, diagnosis and treatment of RA before its progression towards a debilitating stage is imperative for patients. Magnetic resonance imaging (MRI) is reported to be the best clinical imaging technique for the diagnosis of RA, allowing observation of the characteristic inflammation and lesions that are not adequately displayed using conventional radiography. Molecular imaging of specific pathological processes in synovitis would increase the likelihood of early diagnosis, disease staging and monitoring. Any of the molecular actors involved in chronic inflammation, cell proliferation and apoptosis represents putative targets for functionalized imaging probes, thus optimizing the diagnostic capability of clinical imaging techniques [20–23].

Our work is integrated within this clinical and scientific context, by trying to develop molecular tools to image IL-7Rα in vivo as a diagnostic biomarker, and as a marker of response to RA therapy. To achieve this goal, a randomized cyclic heptapeptide phage display library was screened against IL-7Rα, leading to the identification of two peptides specific to this biomarker, one of them being a putative blocking agent for IL-7 (called P725). The peptides were characterized in terms of affinity, biologic activity and diagnostic potential by MRI. The peptide dedicated to MRI applications (called P258) was coupled to ultra-small superparamagnetic particles of iron oxide (USPIO, an MRI contrast agent producing a negative contrast) and its pharmacokinetics, biodistribution and diagnostic potential were evaluated in mice. USPIO present a particular interest for molecular imaging due to their excellent MRI efficacy, biocompatibility and biodegradability. The blood half-life of this material is significantly prolonged by coating it with hydrophilic polymers, such as poly(ethylene glycol) (PEG), which reduce its opsonization and the clearance by the reticuloendothelial system, thus improving the targeting of specific biomarkers [24, 25]. The vectorizing peptide P258 and PEG used as a coating material were covalently coupled to the carboxyl groups exposed at the surface of USPIO.

## Methods

### The experiment of phage display and characterization of the candidate phage clones

#### Phage display library and Escherichia coli host strain

IL-7Rα-targeted peptides were searched within a disulfide-constrained combinatorial library of random linear heptapeptides fused to the minor coat protein (pIII) of M13 bacteriophage (Ph.D.-C7C™ phage display library, New England BioLabs® Inc., Westburg b.v., Leusden, The Netherlands). The *E.coli* host ER2738 (*E. coli* K12 ER2738, F^+^, tetracycline-resistant strain; New England BioLabs®) was used for phage amplification and clone isolation.

#### The panning of phage display library against IL-7Rα

The phage display library was panned against recombinant human IL-7 Rα/Fc chimera (R&D Systems, Abingdon, UK), which was immobilized alternatively on Dynabeads® Protein A or G (Invitrogen Dynal, Merelbeke, Belgium) during the four rounds of biopanning. To increase the peptide specificity and stringency, the target concentration was diminished from 100 nM to 75 nM during the panning rounds; Dynabeads Protein A/Protein G were alternated at each round of panning; the incubation time with the target was reduced stepwise; the incubation times with blocking or related proteins (BSA, Fc-IgG (human IgG, Fc fragment from plasma, Calbiochem, VWR, Leuven, Belgium), fragment 3 of recombinant human FN-1 (R&D Systems)) were increased stepwise, and the Tween-20 concentration was increased at each panning round (i.e., 0.1–0.5 %) in the incubation and rinsing buffer (phosphate-buffered saline, PBS; per liter: 8 g NaCl, 0.2 g KCl, 2.31 g Na_2_HPO_4_ × 12 H_2_O, 0.2 g KH_2_PO_4_, pH 7.4). The detailed bio-panning protocol is available in Additional file 1: Methods.

#### Evaluation of the affinity of the phage clones for IL-7Rα

For affinity tests, IL-7Rα was diluted at a concentration of 10 μg/mL in 0.1 M NaHCO_3_, pH 8.6, and immobilized overnight at 4 °C. The control wells were coated with 5 mg/mL of BSA or with 10 μg/mL of FN and used to evaluate the phage non-specific binding. Phages were diluted in PBS supplemented with 0.5 % Tween-20 (PBST), and were incubated with both test and control wells (2 h, 37 °C). Bound phages were detected with horseradish peroxidase (HRP)-conjugated anti-M13 antibody (Amersham Pharmacia Biotech Benelux, Roosendaal, The Netherlands) diluted 1:5000 in THBS completed with 5 mg BSA/mL. The staining reaction was developed with 2,2´-Azino-bis(3-Ethylbenzothiazoline-6-sulfonic acid) (ABTS), diamonium salt (Sigma-Aldrich, Bornem, Belgium) solution completed with 0.05 % H_2_O_2_. The optical density (OD)_405_ was measured using a microplate reader (StatFax-2100, Awareness Technology, Fisher Bioblock Scientific, Tournai, Belgium).

To confirm the binding specificity of three selected clones, immobilized IL-7Rα was pre-incubated with a range of human recombinant IL-7 (R&D Systems) concentrations. The phages were added 30 minutes later at a concentration equal to their apparent dissociation constant (K*_d_) and the incubation was continued for one more hour. Phages bound to the target were detected with HRP-conjugated anti-M13 antibody as described above. The affinity tests are additionally described in Additional file 1: Methods.

#### Sequencing of the phage clones

Phage DNA was isolated and purified by phenol extraction–ethanol precipitation. The sequence reaction (Mastercycler Personal) was carried out by the Sanger method, using the Quick Start Kit (Beckman Coulter^TM^, Analis, Namur, Belgium) and a 20-base primer (5’-CCCTCATAGTTAGCGTAACG-3’) (New England Biolabs). Sequence reading was performed with JaMBW 1.1 software (http://bioinformatics.org/JaMBW/), and peptide sequences were aligned with the basic local alignment search tool (BLAST).

### Characterization of the selected IL-7Rα-binding peptides

Three peptides were selected for synthesis and subsequent evaluation. They were synthesized as biotinylated or not biotinylated 8-amino-3,6-dioxaoctanoyl derivatives and encoded as follows: P722 (clone 36/R2: C-PHPQRPA-C), P725 (clone C29/R3: C-KIMKSMP-C) and P726 (clone C37/R2: C-ASACPPH-C). Peptide 726 was also synthetized in a linear version (ASACPPH) and was encoded as P258.

#### Estimation of the apparent dissociation constant (K*_d_)

Serial dilutions of biotinylated peptides were incubated with IL-7Rα or FN immobilized on ELISA plates as described above and blocked with protein-free blocking buffer (PFBB) (Perbioscience, Erembodegem, Belgium). Peptides bound to the target were detected with a goat anti-biotin antibody, followed by a peroxidase-conjugated anti-goat antibody made in horse (both from Vector Labconsult, Brussels, Belgium). The staining reaction was developed with ABTS/H_2_O_2_, and OD_405_ was measured with a microplate reader. The protocol is largely described in Additional file 1: Methods.

#### Estimation of the half maximal inhibitory concentration (IC_50_)

The K*_d_ of IL-7 was determined by incubating a range of IL-7 concentrations with immobilized IL-7Rα. The bound IL-7 was then detected with a polyclonal goat anti-human IL-7 antibody (R&D Systems) followed by a horse anti-goat antibody coupled to HRP (Vector Labconsult). To estimate the IC_50_ of peptides, they were pre-incubated with IL-7Rα-coated wells in a range of concentrations; IL-7 was then added at a concentration equal to its K*_d_ and the incubation continued for another 90 minutes. IL-7 bound to the target was detected as described. The detailed protocol is available in Additional file 1: Methods.

#### Binding of peptides to Jurkat cells and co-localization with IL-7Rα by immunofluorescence

Jurkat cells were cultured in RPMI-1640 culture medium supplemented with 10 % newborn calf serum heat-inactivated and 1 % antibiotic-antimycotic (all from Invitrogen). The cells were stimulated with 0.4 μM of 5-Aza-2’-deoxycytidine (ADC, Sigma-Aldrich) [26]. The stimulated cells were immobilized on poly-L-lysine (Sigma-Aldrich) coated coverslips and fixed in a solution of 4 % formaldehyde (Sigma-Aldrich). After blocking the cells with PFBB, they were co-incubated with mouse anti-human IL-7Rα monoclonal antibody (Sigma-Aldrich) and biotinylated peptides P258 (0.04 μM) or P725 (5 μM). Biotinylated peptides were detected with a goat anti-biotin antibody followed by fluorescein-conjugated rabbit anti-goat antibody (both from Vector Labconsult). Anti-IL-7Rα antibody was detected with Texas Red horse anti-mouse antibody (Vector Labconsult). Control samples consisted of ADC-stimulated or non-stimulated (NS) Jurkat cells incubated with low-affinity peptide binders (e.g., P255, P259), a mouse IgG isotype control, or with secondary antibodies after excluding the primary antibodies. The coverslips were mounted on slides with Vectashield mounting medium for fluorescence with 4',6-diamidino-2-phenylindole (DAPI) (Vector Labconsult) and observed on a DM2000 Leica microscope equipped with a DFC 425C camera (Leica Microsystems, Groot Bijgaarden, Belgium). The protocol is additionally described in Additional file 1: Methods.

#### Immunohistochemical detection of biotinylated P258 and of IL-7Rα in knee biopsy samples from patients with RA

Synovial knee biopsies were obtained by needle arthroscopy in three untreated patients with active RA. Fresh biopsy samples (between four and six per patient) were fixed overnight in 10 % formalin buffer at pH 7.0 and embedded in paraffin. Sections of 5 μm thickness were cut and treated to remove paraffin (two baths of toluene for 10 minutes each) and rehydrate them (three baths of 95 % ethanol for 10 minutes each, followed by 10 minutes in running cold tap water and 5 minutes in distilled water), followed by unmasking of epitopes in a citrate solution according to standard procedures. The endogenous peroxidases were blocked with 0.3 % H_2_O_2_ in PBS and the non-specific epitopes were blocked with PFBB.

The tissue sections were incubated with mouse anti-human IL-7Rα monoclonal antibody (Sigma-Aldrich), followed by a peroxidase-conjugated monoclonal anti-mouse antibody produced in goat (Sigma-Aldrich). The sections were then stained with 0.05 % 3,3'-diaminobenzidine (DAB) completed with 0.02 % H_2_O_2_ in PBS. Finally, they were counterstained with Hemalun and Luxol fast blue and mounted in a permanent medium.

Endogenous biotin was blocked with a blocking kit (Invitrogen) before incubating the histologic sections with 1 μM of P258 (or with control peptides P255 and P259), followed by a goat anti-biotin antibody and a peroxidase-conjugated anti-goat antibody made in horse (both from Vector Labconsult). The staining and counterstaining were performed as described for anti-IL-7Rα monoclonal antibody. Additional information is available in Additional file 1: Methods. The study was approved by the Ethical Committee of the Université Catholique de Louvain, and informed consent was obtained from all patients.

#### Phospho-STAT5 detection on Jurkat cells and modulation by peptide P725

The experiment was performed in triplicate on ADC stimulated and NS cells distributed in several culture tubes treated with different compounds diluted in the culture medium as follows: (A) cells treated for 48 h with IL-7 (eBioscience, Vienne, Austria); (B) cells pre-incubated for 30 min with P725; (C) cells pre-incubated for 30 min with mouse anti-human IL-7Rα monoclonal antibody (Sigma-Aldrich). IL-7 was then added in samples B and C, and incubation continued for 48 h. The solutions were replaced every day after culture tube centrifugation and supernatant removal.

The treated cells were rinsed and immobilized on poly-L-lysine-coated coverslips. They were fixed with 4 % formaldehyde and 100 % methanol, and then blocked with a solution of 5 % normal goat serum and 0.3 % Triton X-100 prepared in PBS. Subsequently, the cells were co-incubated with human Phospho-STAT5 (Tyr694 D47E7 XP®) antibody made in rabbit and anti-Pan-keratin (C11) antibody made in mouse (both from Bioké, Leiden, The Netherlands). The cells were subsequently co-incubated with fluorescein-conjugated goat anti-rabbit antibody and horse Texas Red-conjugated anti-mouse antibody (both from Vector Labconsult). The coverslips were finally mounted on slides with Vectashield mounting medium for fluorescence with DAPI (Vector Labconsult). Detailed protocols are available in Additional file 1: Methods.

The signal intensity (SI) was measured on microphotographs corresponding to three to six different microscopic fields using the ImageJ software (National Institutes of Health, USA), and the relative ratio of fluorescent labeling (RRFL) was then calculated using the subsequent equation:

$$ RRFL\kern0.5em =\kern0.5em \left(\frac{S{I}_{sample}}{Number\kern0.5em  of\kern0.5em  cells}\right)/\left(\frac{S{I}_{negative\kern0.5em  controls}}{Number\kern0.5em  of\kern0.5em  cells}\right)\kern0.5em \times \kern0.5em 100 $$

#### Evaluation of the lysosome content of Jurkat cells

For lysosome tracking, Jurkat cells stimulated and treated as described above were incubated with a solution of Hoechst 33342 (for nuclei staining), followed by Lysotracker® Red DND-99 (Image-mITT LIVE lysosomal and nuclear labeling kit, Life technologies, Merelbeke, Belgium). The cells were then mounted between microscope slides and coverslips, observed under a microscope and the RRFL was evaluated as described above . The protocol is additionally described in Additional file 1: Methods.

### Preparation and characterization of the imaging probe

#### Synthesis and characterization of USPIO-P258

Peptide P258 was synthesized by the company PolyPeptide (Strasbourg, France) as an 8-amino-3,6-dioxaoctanoyl (two PEG units, used as linker) derivative (PolyPeptide, Strasbourg, France), the C-terminus of the peptide being amidated. It was then covalently conjugated to USPIO as previously described [24]. The vectorized nanoparticles (USPIO-P258) were rendered stealth by a PEG coat, which was also used to prepare the non-specific nanoparticles (USPIO-PEG). The transverse relaxivity, r_2_, evaluated at 37 °C and 60 MHz in mouse blood plasma was of 98.94 s^−1^mM^−1^ for USPIO-P258 and of 107.75 s^−1^mM^−1^ for USPIO-PEG. The hydrodynamic diameter of USPIO-P258 was measured by photon correlation spectroscopy (PCS, Brookhaven system BI-160 (New York, USA) equipped with a He-Ne laser (=633 nm, 35 mW)) after 2 h of incubation at room temperature in water (48 nm), PBS (40 nm) and RPMI culture medium (37 nm).

The K*_d_ of USPIO-P258 for the binding to IL-7Rα was determined by ELISA, using a rabbit anti-PEG monoclonal antibody (Abcam, Cambridge, UK), biotinylated goat anti-rabbit IgG and Vectastain ABC kit (both from Vector Labconsult) as previously described [25]; the IC_50_ of USPIO-P258 was evaluated using the same method as that described previously. The eventual cytotoxic effects of USPIO-P258 were determined on HepaRG™ cell line (Life Technologies) using the MTT assay (in vitro toxicology assay kit MTT-based, Sigma-Aldrich) as previously described [25].

#### Evaluation of USPIO-P258 binding to Jurkat cells

ADC-stimulated or NS Jurkat cells were incubated (*n* = 3/experimental group) with USPIO-P258 or USPIO-PEG at an iron concentration of 1–3 mM. The cells were subsequently rinsed and mineralized in acidic conditions, and iron concentration in cell samples was determined by relaxometry on a Bruker Minispec Mq60 (Karlsruhe, Germany) based on a calibration curve. For magnetic resonance imaging (MRI) analysis and the measurement of the transverse relaxation time (T_2_), the cells incubated with contrast agents were suspended in 2 % gelatin prepared in PBS. The MR images were acquired on a 300 MHz (7 T) Bruker Biospec imaging system (Ettlingen, Germany). A T_2_-weighted Rapid Acquisition with Relaxation Enhancement (RARE) sequence was used for cell visualization. The T_2_ was measured with a Multi-Slice-Multi-Echo (MSME) sequence. The results were expressed as transverse relaxation rates (R_2_ = 1/T_2_) normalized to gelatin (R_2_^Norm^) and measured in s^−1^.

USPIO-P258 was co-localized with IL-7Rα expressed by Jurkat cells using an immunofluorescence protocol. At the end of the MRI studies, the cells were recovered from gelatin, resuspended in PBS and immobilized on poly-L-lysine coated coverslips, before being fixed and blocked as described previously. The cells were then co-incubated with mouse anti-human IL-7Rα monoclonal antibody (Sigma-Aldrich) and with rabbit monoclonal anti-PEG antibody (Bio-Connect/Epitomics, Huissen, The Netherlands). The cells were subsequently co-incubated with Texas Red horse anti-mouse antibody and fluorescein goat anti-rabbit antibody (both from Vector Labconsult). The coverslips were finally mounted on slides with a mounting medium for fluorescence (Vector Labconsult). The RRFL was evaluated as described above. The protocols are detailed in Additional file 1: Methods.

#### Evaluation of pharmacokinetic parameters, biodistribution and diagnostic ability of USPIO-P258 ﻿by molecular imaging

The experiments performed on animals were approved by the Ethics Committee of the University of Mons and they comply with the Directive 2010/63/EU. For pharmacokinetics and biodistribution evaluation, NMRI mice (*n* = 3 per time point; Harlan, Horst, The Netherlands) were injected with USPIO-P258 at 100 μmol Fe/kg body weight (b.w.). The pharmacokinetics and biodistribution of USPIO-PEG, used as a control contrast agent, were previously determined [25]. The negative control animals were left untreated. The blood, urine and organs (kidneys, liver, spleen and lungs) were collected at various time intervals after the injection of the contrast agent, and R_2_ was measured on a Bruker Minispec mq60. Several pharmacokinetic parameters (the elimination half-life (T_e1/2_), the volume of distribution steady state (VD_ss_) and the total clearance (Cl_tot_)) were calculated after measuring the blood concentration of contrast agents [24, 25].

For molecular imaging studies, autoimmune RA was induced in DBA/1 male mice (*n* = 4 per experimental group) aged 10 weeks (Harlan), by immunization with an emulsion of complete Freund’s adjuvant and type II collagen from chicken sternal cartilage (Sigma-Aldrich) as described [27]; the healthy control mice were left untreated (*n* = 4 per experimental group). The mice were distributed into four groups of four mice (two CIA and two healthy groups) and were injected intravenously (i.v.) in the caudal vein at a dose of 100 μmol Fe/kg b.w. with either USPIO-P258 or USPIO-PEG for MRI studies; the mice not injected with contrast agents were used as controls for relaxometric studies. The acquisition of images started immediately after contrast agent injection with RARE or 3D fast imaging with steady-state precession (FISP) sequences.

Signal intensity (SI) values for each time point were measured on RARE images within regions of interest (ROIs) drawn manually at the level of the paw using the ImageJ image analysis software (National Institutes of Health, USA). The standard deviation (SD) of the noise was measured in a region situated out of the animal’s image. SI enhancement (ΔSNR%) was calculated for each sagittal or coronal image according to the following equation:

$$ \varDelta \kern0.22em SNR\%=\frac{\left(S{I}_{post}/ Noise\;SD\right)-\left(S{I}_{pre}/ Noise\;SD\right)}{\left(S{I}_{pre}/ Noise\;SD\right)}\times 100 $$

where SNR = signal-to-noise ratio, SI_post_ = post-contrast SI, and SI_pre_ = pre-contrast SI.

Technical details are presented in Additional file 1: Methods.

The total area (TA) occupied by black pixels in pre-contrast and 108 minutes post-contrast (thresholded to the pre-contrast level) RARE images of the hind limbs of CIA mice injected with USPIO-P258 was evaluated using the ImageJ software as previously described [24]. Then, the percentage difference of TA (%DTA) in post-contrast as compared to the pre-contrast images was calculated.

#### Biodistribution, IL-7Rα expression, Perls’-DAB staining of USPIO derivatives on paw samples and Masson’s Trichrome staining; immunofluorescent co-localization of USPIO-P258 with IL-7Rα

At the end of the MRI studies, the mice were killed by a lethal dose of sodium pentobarbital (600 mg/kg b.w., intraperitoneal (i.p.)) and paws were harvested for (immuno)histochemical and biodistribution studies after transcardial perfusion with PBS. Biodistribution in freshly sampled paws was evaluated as described previously, by measuring the R_2_ on a Bruker Minispec mq60 working at 60 MHz and 37 °C. For (immuno)histochemical studies, paws were fixed in buffered paraformaldehyde and decalcified in Biodec-R (Bio-optica Milano s.p.a) for 8 days before paraffin embedding. IL-7Rα was detected with mouse anti-IL-7Rα monoclonal antibody and peroxidase-conjugated monoclonal anti-mouse antibody produced in goat (both from Sigma-Aldrich) as described previously. USPIO derivatives were detected on paw sections by histochemical analysis, using the Perls’-DAB iron staining protocol [24, 28], while the paw morphology was studied after Masson’s trichrome staining using the Accustain® kit (Sigma-Aldrich) as previously described [24, 25].

USPIO derivatives were also co-localized with IL-7Rα-expressing cells by co-incubating paw sections with rat anti-PEG (Abcam) and mouse anti-IL-7Rα (Sigma-Aldrich) monoclonal antibodies, followed by Texas red-conjugated goat anti-rat and Fluorescein-conjugated horse anti-mouse antibodies (both from Vector Labconsult). The protocols are additionally described in Additional file 1: Methods.

The TA occupied by IL-7Rα staining on the paw sections of CIA mice injected with USPIO-P258 was then quantified on microphotographs using the ImageJ software. The coefficient of correlation *r* ^2^ was calculated and the %DTA obtained after analyzing the MR images; these two parameters were also correlated with the severity score of CIA as determined on living mice according to the definitions provided by Brand et al. [27].

### Statistical analysis

The results are expressed as means ± SD. One-way analysis of variance (ANOVA), performed with SigmaPlot 11.0 software, was applied to compare different experimental groups. For the groups where the equal variance test was not satisfied, the statistical significance was furthermore confirmed after applying the Holm-Sidak and Bonferroni tests. Results were considered statistically significant at *p* < 0.05.

## Results

### Identification and characterization of the candidate phage clones

A disulfide constrained heptapeptide phage display library was screened against the extracellular domain of IL-7Rα (E^21^–D^239^), which contains an FN type III repeat-like sequence (A^131^–I^231^). To remove the potential binders to FN type III domain, the phage library was pre-selected against FN before being screened against IL-7Rα. After four rounds of selection, the affinity of the phage pools towards IL-7Rα was evaluated and compared to that for BSA, used as a blocking agent (Additional file 2: Figure S1A and B). The results show that the second and third rounds of panning present an optimal binding to IL-7Rα, whereas the fourth round had lost its affinity. We therefore isolated 102 clones issued from the second and third rounds of panning, and the K*_d_ against IL-7Rα and FN was determined. Among them, 12 clones (9 from the second round and 3 from the third round) presented the highest affinity for IL-7Rα, with K*_d_ values that ranged from 0.21 nM to 4.15 nM (Additional file 2: Figure S1C and D). The K*_d_ of their binding to FN was variable and ranged between 99.5 μM and 1.98 nM. Based on their K_d_^FN^/K_d_^IL-7Rα^ ratio (477, 163 and 6 for the strongest binders), three clones (36/R2, 29/R3 and 37/R2) were selected for supplemental characterization (see Additional file 2: Figure S2 for individual K*_d_ values).

The binding specificity was furthermore validated for these three clones by competing them with IL-7. The IC_50_ of IL-7 estimated in these conditions provides information on the amount of IL-7 required to dissociate phages from their binding sites. In other words, the higher IC_50_ value, the stronger binder is the phage clone candidate. We have therefore identified an IC_50_ value of 2.41 × 10^−6^ M, 3.98 × 10^−8^ M and 5.10 × 10^−6^ M for the competition between IL-7 and the three selected clones (Table 1; Additional file 2: Figure S2).

**Table 1:** Alignment of peptides expressed by clones 29, 36 and 37 with relevant protein sequences (identified with Swiss-Prot accession numbers), where the homologous amino acids are shown

Peptide (IL-7 IC_50_)	Homology
C36/R2: C-PHPQRPA-C (2.41 × 10^−6^ M)	Suppressor of cytokine signaling 7 (O14512): ^92^ **P**Q**PQ**P**PA** ^98^; Signal transducer and activator of transcription 4 (Q14765): ^327^ **﻿HPQRP** ^331^; Fibronectin (P02751): ^298^ **PHPQ**P**P** ^303^; Serine/threonine-protein kinase ICK (Q9UPZ9): ^591^ **PHP**G**RP** ^596^
C29/R3: C-KIMKSMP-C (3.98 × 10^−8^ M)	Fibronectin type III domain-containing protein 3B (Q53EP0): ^1169^﻿**MKSM** ^1172^; Serine/threonine-protein kinase ATR (Q13535): ^75^ **﻿IMKS**S**P** ^80^; Serine/threonine-protein kinase mTOR (P42345): ^1701^ **MK**N**M**---**KI**L**K**N**M** ^1998^; Interferon omega-1 (P05000): ^171^ **IMKS**L^175^; Transmembrane and immunoglobulin domain-containing protein 1 (Q6UXZ0): ^245^ **KIMK** ^248^; Tumor necrosis factor alpha-induced protein 8-like protein 3 (Q5GJ75): ^164^ **KIMK** ^167^; Tyrosine-protein kinase STYK1 (Q6J9G0): ^343^ **KIMK**---**IMKS** ^361^; Frizzled-9 (O00144): ^429^ **KIMK** ^432^; Cadherin-16 (O75309): ^777^ **MK**G**MP** ^781^
C37/R2: C-ASACPPH-C (5.10 × 10^−6^ M)	Tumor necrosis factor receptor superfamily member 27 (Q9HAV5): ^43^ **ACPP** ^46^; Cadherin EGF LAG seven-pass G-type receptor 3 (Q9NYQ7): ^1955^ **﻿CPPH** ^1958^; Rho GTPase-activating protein 29 (Q52LW3): ^1212^ **ASACP** ^1216^; Serine/threonine-protein kinase WNK2 (Q9Y3S1): ^688^ **﻿A**P**ACPP**---**AS**P**CP**---**PP** ^1741^; Serine/threonine-protein kinase D2 (Q9BZL6): ^860^ **ACPP**Q^864^; Phosphoserine phosphatase (P78330): ^186^ **ACPP** ^189^; Laminin subunit alpha-1 (P25391): ^276^ **AS**S**CP**---**CPPH** ^1019^; Ephrin type-A receptor 3 (P29320): ^290^ **CPPH** ^293^; Ephrin type-A receptor 4 (P54764): ^293^ **CPPH** ^296^; Ephrin type-A receptor 5 (P54756): ^322^ **CPPH** ^325^; Ephrin type-A receptor 6 (Q9UF33): ^296^ **CPPH** ^299^; Ephrin type-A receptor 8 (P29322): ^293^ **CPPH** ^296^

The aligned amino acids are highlighted as bold. The half maximal inhibitory concentration (IC_50_) values of IL-7 in competition with the same phage clones are also shown

The peptide sequence of the 12 initial candidate phage clones was deciphered (Additional file 2: Figures S1C and 1D) and their analysis revealed a high frequency of basic (His, Lys) and alcohol (Ser) amino acids, but also of Pro. The alignment of the three best peptide clones with relevant human proteins was searched using the basic alignment search tool BLAST (Table 1), and interesting homologies were found with several serine/threonine-protein kinases, with adhesion molecules and proteins of the extracellular matrix or involved in cytoskeletal organization, cell migration, embryogenesis and inflammation.

### Synthesis and in vitro characterization of the candidate molecular imaging peptides

**Fig. 1:**
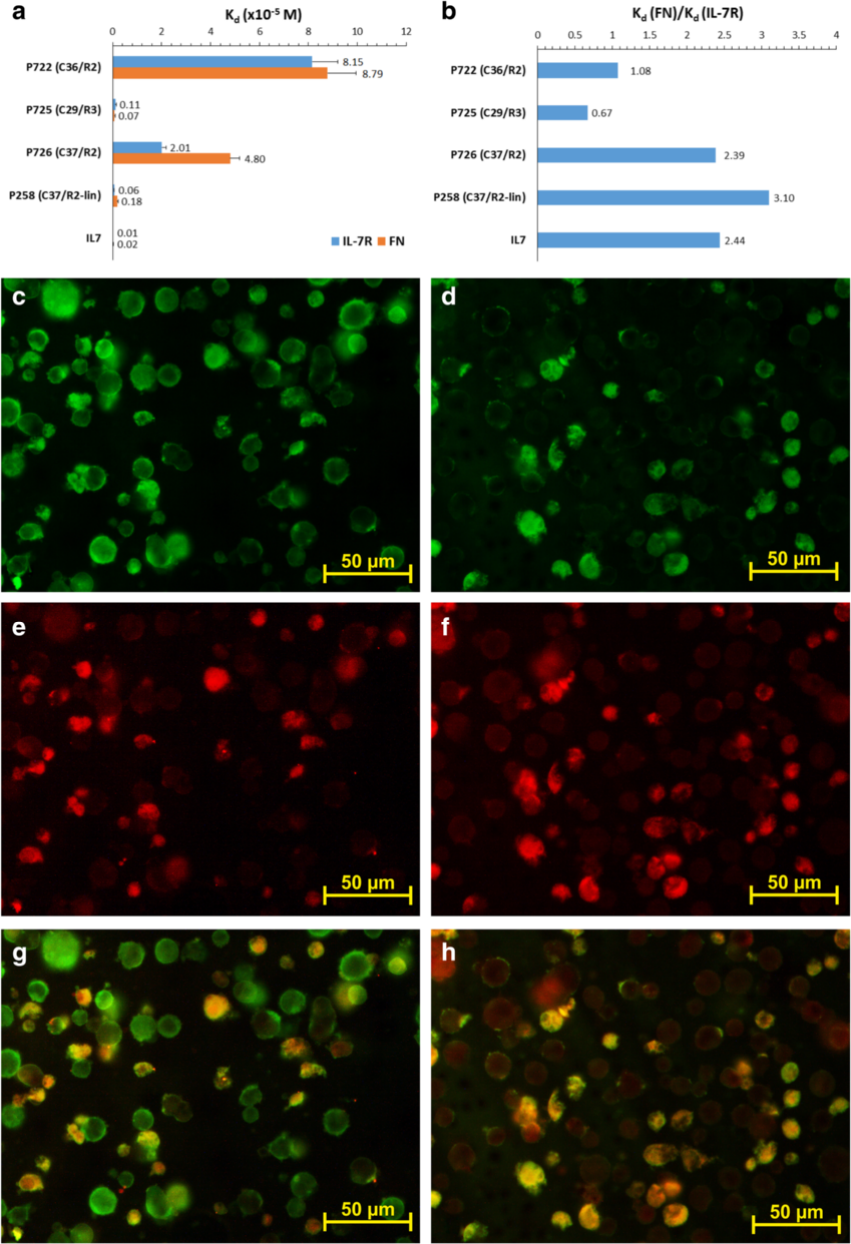
Apparent dissociation constant (*K*_*d*_) values of the synthesized selected peptides and of IL-7 were determined for their binding to IL-7 receptor (IL-7R) and fibronectin (*FN*) (**a**). The ratio between K_d_ values for FN over IL-7R binding are shown (**b)**. The binding of peptides P725 (**c**) and P258 (**d**) to Jurkat cells stimulated by 5-Aza-2′-deoxycytidine was compared to anti-IL-7R antibody (**e** and **f**), which co-localizes with peptides as shown by their superposition (**g** and **h**). Peptide annotation: *P722* C-PHPQRPA-C, *P725* C-KIMKSMP-C, *P726* C-ASACPPH-C, *P258* ASACPPH

The three candidate peptides were synthesized first as biotinylated derivatives and their K*_d_ for IL-7Rα and FN binding was evaluated (Fig. 1a; Additional file 2: Figure S3); peptide expressed by clone 37/R2 was also synthesized in a linear version by removing the Cys-constraint. Among the four synthesized peptides, P725 (expressed by clone 29/R3; K*_d_ of 0.11 × 10^−5^ M) and P258 (the linearized peptide expressed by clone 37/R2; K*_d_ of 0.06 × 10^−5^ M) presented the highest affinity for IL-7Rα, but the ratio K*_d_^FN^/K*_d_^IL-7Rα^ revealed a specific binding to IL-7Rα in the case of P258 (Fig. 1b). This was even higher than that of IL-7 and their K*_d_^IL-7Rα^ was equivalent. Taken together, these properties emphasize P258 as the best candidate for molecular imaging of RA after its conjugation to an imaging probe. At the same time, the IC_50_ value of P725 was of 1.64 × 10^−5^ M (Additional file 2: Figure S3), highlighting it as an optimal competitor of IL-7. However, its equivalent affinity for IL-7Rα and FN renders this peptide inadequate for molecular imaging; the ubiquitous FN expression could generate a high potential background.

Peptides P258 and P725 (Additional file 2: Figure S4A and B) were thus selected for further characterization with the aim of developing *in vivo* imaging (P258) and blocking (P725) applications. The theoretical biochemical parameters (Table 2) show that peptide P258 has a half-life of 4.4 h as predicted by the N-end rule [29], while that of P725 is 1.2 h. The N-end rule stipulates that proteasome degradation of proteins within cells depends on the ubiquitin covalent conjugation at the N-terminus residue of the protein to be hydrolyzed. The primary destabilising residues are positively charged (i.e., lysine, arginine, histidine), whereas methionine is a stabilizing residue. The isoelectric point (pI) value and hydrophilic properties, suggested by LogP, LogD, GRAVY and aliphatic index, indicate that both peptides are ionized at physiological pH and may be able to interact with charged residues on the targeted protein.

**Table 2:** Theoretical biochemical parameters of peptide P725 and P258 as estimated by using the ExPASy proteomics server, proteomics and sequence analysis tools

Parameter	Peptide P725	Peptide P258
Half-life	1.2 h	4.4 h
pI	8.90	6.78
LogP (of ionic species)	−4.49	−7.66
LogP (of non-ionic species)	−2.65	−0.31
LogD (at pH 7.4)	−4.46	−4.58
GRAVY	0.344	−0.157
Aliphatic index	43.33	28.57

LogP and LogD were calculated by using the MarvinSketch 5.11.5 software (2013, http://www.chemaxon.com). Calculator plugins were used for structure property prediction and calculation. Half-life was theoretically estimated in mammalian reticulocytes *in vitro*. Aliphatic index is the relative volume occupied by aliphatic side chains. *pI* isoelectric point, *LogP* partition coefficient, *LogD* distribution coefficient estimated at pH 7.4 and a salt concentration of 150 mM, *GRAVY* grand average of hydropathicity (predicts the hydrophobicity)

The two peptides were co-localized with IL-7Rα expressed by ADC-stimulated Jurkat cells (Fig. 1c-h) as proven by the yellow/orange color obtained after overlapping the green staining of peptides (Fig. 1c and d) with the red staining of IL-7Rα (Fig. 1e and f), attesting for their specific interaction. Intensity of the IL-7Rα staining using a monoclonal antibody was decreased by 57 % in the presence of P725, thereby suggesting that this peptide competed with the antibody binding sites on the receptor. Control samples were characterized by very weak staining or no staining (data not shown), which contributed to the validation of specific cell binding of the selected peptides.

### In vitro characterization of the functionalized imaging probe USPIO-P258

Peptide P258 was retained for the development of vectorized contrast agents. We confirmed by immunohistochemistry that P258 stains synovial tissue from patients with RA (Additional file 2: Figure S5); the weak staining produced by control peptides P255 and P259 (data not shown) attested for the specific binding of P258 to IL-7R. After grafting to USPIO-258 (Additional file 2: Figure S6A), the K*_d_ of the vectorized contrast agent was 4.9 × 10^−6^ M for the binding to IL-7Rα (Additional file 2: Figure S6B); with USPIO-P258 there was no binding to the PFBB-coated ELISA plate, proving its specific interaction with the target. With the negative control contrast agent, USPIO-PEG, there was negligible binding to IL-7Rα at high concentrations. The IC_50_ of USPIO-P258 was of 6.6 × 10^−6^ M, suggesting that functionalized nanoparticles may have the ability to dislocate IL-7 from IL-7Rα (Additional file 2: Figure S6C). The results of the MTT assay demonstrated that USPIO-P258 did not produce cytotoxic effects at either of the concentrations or times of incubation tested in our experimental conditions (Additional file 2: Figure S6D).

**Fig. 2:**
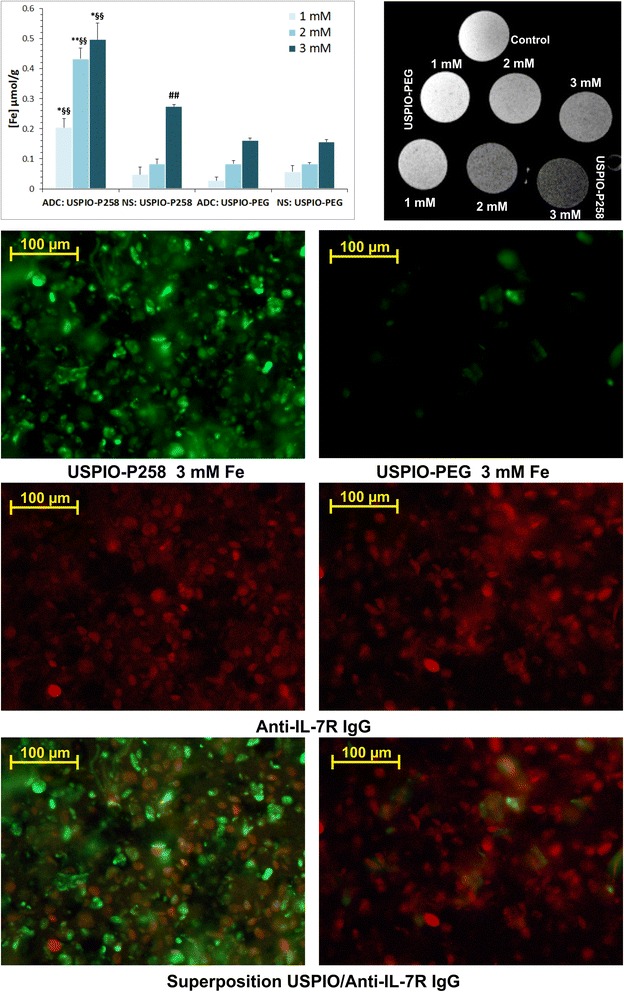
The binding of ultra-small superparamagnetic particles of iron oxide (USPIO)-P258 and of USPIO-poly(ethylene glycol) PEG (*USPIO-PEG*) to Jurkat cells stimulated by 5-Aza-2′-deoxycytidine (*ADC*), or not stimulated (*NS*), is presented as Fe concentration (*left histogram*). The binding of these contrast agents to ADC-stimulated Jurkat cells is also exemplified by the magnetic resonance image (*right upper column*); *control* represents Jurkat cells not incubated with contrast agents and included in gelatin. USPIO-P258 and USPIO-PEG bound to ADC-stimulated Jurkat cells were detected by immunofluorescence with anti-PEG antibody (*green* fluorescence) and co-localized with anti-IL-7 receptor (*anti-IL-7R*) antibody (*red* fluorescence) on the same cells. *p < 0.05, **p < 0.01 for ADC vs. NS; ^§§^ *p* < 0.01 for USPIO-P258/ADC vs. USPIO-PEG/ADC; ^##^ *p* < 0.01 for USPIO-P258/NS vs. USPIO-PEG/NS

The binding of USPIO-P258 to ADC-stimulated Jurkat cells was evaluated and compared to USPIO-PEG or to NS Jurkat cells. The measurement of iron concentration was significantly higher in ADC samples incubated with USPIO-P258 as compared to NS samples or cells incubated with USPIO-PEG (Fig. 2). At high iron concentrations (i.e., 3 mM corresponding to 2.73 × 10^−7^ M nanoparticles by assuming approximately 11,000 iron atoms per particle [24]), USPIO-P258 was also captured by NS cells (*p* < 0.01 vs. USPIO-PEG), but this may be explained by the constitutive level of IL-7R expression. Iron concentration measured on cell samples was then converted into the number of USPIO-P258 particles bound per cell. We have thereby obtained about 3 × 10^4^ to 7.6 × 10^4^ particles/ADC cell and about 7.3 × 10^3^ to 4.2 × 10^4^ particles/NS cell (Additional file 2: Figure S6E). These estimations are in agreement with the data published by other authors with regard to the number of IL-7R molecules expressed by different types of T cells [30].

The ability of USPIO-P258 to bind ADC-stimulated cells was validated by MRI and the R_2_^Norm^ measurement, but also by immunofluorescence that demonstrated co-localization of USPIO-P258 (but not of USPIO-PEG) with IL-7Rα expressed by ADC cells (Fig. 2; Additional file 2: Figures S7A, B). There was significant correlation between R_2_^Norm^ measured by MRI and RRFL measured by immunofluorescence (Additional file 2: Figure S7C), attesting for the specific binding of USPIO-P258; IL-7Rα expression was about six times higher in ADC cells vs. NS cells (Additional file 2: Figure S7D).

### In vivo characterization of the functionalized imaging probe USPIO-P258

USPIO-P258 has faster blood clearance as compared to USPIO-PEG, as demonstrated by the T_e1/2_ (118 minutes vs. 284 minutes for UPIO-PEG) and Cl_tot_ (0.595 ml/min/kg vs. 0.116 ml/min/kg for USPIO-PEG). The VD_ss_ of USPIO-P258 (0.1 L/kg) is about two times larger than that of USPIO-PEG (0.0475 L/kg), which is confined to the vascular space (Table 3; Additional file 2: Figure S7E). USPIO-P258 was found in urine at concentrations six to eight times higher than USPIO-PEG (Additional file 2: Figure S7F), the highest being observed at 120 minutes post injection.

**Table 3:** Pharmacokinetic parameters of USPIO-P258 in comparison to USPIO-PEG, as determined in healthy NMRI mice

Pharmacokinetic parameters	USPIO-P258	USPIO-PEG
T_e1/2_ (minutes)	118	284
VD_ss_ (L/kg)	0.100	0.0475
Cl_tot_ (mL/min/kg)	0.595	0.116

*USPIO* ultra-small superparamagnetic particles of iron oxide, *PEG* poly(ethylene glycol), *T*_*e1/2*_ elimination half-life, VD_ss_ volume of distribution steady state_,_ Cl_tot_ total clearance

The biodistribution of USPIO-P258 was evaluated by a bi-exponential treatment of T_2_ relaxation curves, which provided two relaxation components of the water protons, i.e., a fast component (R_2–1_) and a slow one (R_2–2_). USPIO-P258 content in the kidney decreased over time, concomitantly with the increase of urine concentration (Additional file 2: Figures S7G-H). USPIO-PEG also seems to be excreted, at least partly, via the renal system, but its urinary excretion is delayed in comparison with USPIO-P258. The other organs (liver, spleen, lungs) seem only to be transited by USPIO-P258 via the circulatory system, with no significant accumulation (Additional file 2: Figures S8A-F), as opposed to USPIO-PEG, which seems to accumulate in liver and lungs.

**Fig. 3:**
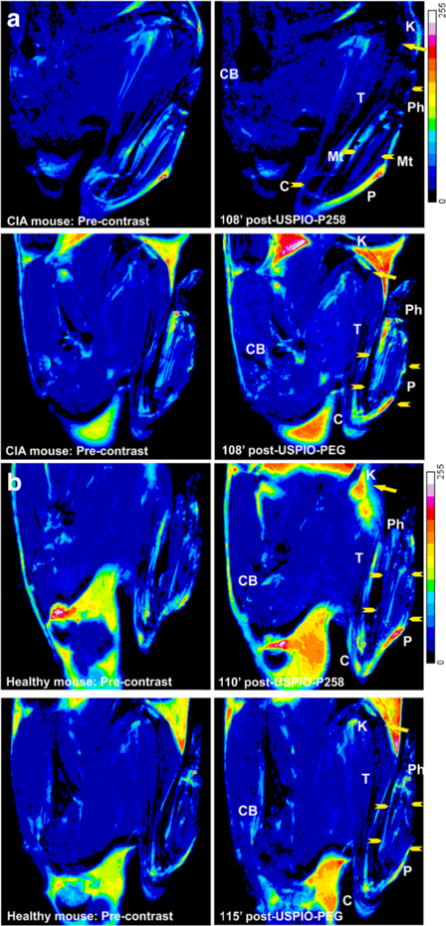
Color overlay of rapid acquisition with relaxation enhancement (RARE) magnetic resonance images (spatial resolution = 156 × 90 μm) of the hind limbs of mice with collagen-induced arthritis (*CIA*) (**a**) or healthy mice (**b**) injected with either ultra-small superparamagnetic particles of iron oxide (*USPIO*)-P258 or USPIO-poly(ethylene glycol) (*USPIO-PEG*) in pre-contrast and about 2 h post contrast. *Arrowheads* indicate the hind paw; *arrow* indicates the knee. *Color overlays* are related to the negative signal enhancement, which is observed by the shift of colors from *blue* to *black*; on the post-USPIO-P258 image of the CIA mouse, *arrowheads* and *arrow* indicate the negatively contrasted constituents of the hind limb. *C* calcaneus, *CB* coxal bones, *K* knee, *P* paw, *Ph* phalanx, *Mt* metatarsal bones, *T* tibia

**Fig. 4:**
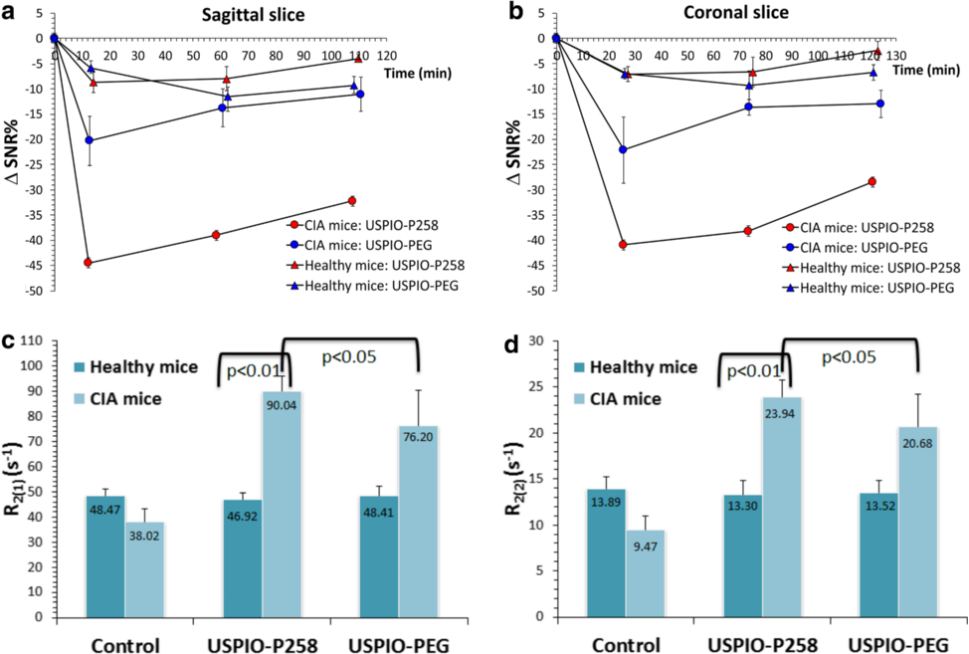
Signal enhancement (% difference in signal-to-noise ratio (Δ*SNR%*)) measured on sagittal (**a**) and coronal (**b**) magnetic resonance images of the paw in mice with collagen-induced arthritis (*CIA*) and healthy mice injected with ultra-small superparamagnetic particles of iron oxide (*USPIO*)-P258 or USPIO-poly(ethylene glycol) (*USPIO-PEG*): *n* = 4 per experimental group; *p* < 0.01 for CIA mice injected with USPIO-P258 as compared to all control groups, except for the coronal slices acquired at 26 minutes post contrast and compared to USPIO-PEG, for which *p* < 0.05. For transverse relaxation rates (*R*_*2*_), R_2(1)_ (**c**) and R_2(2)_ (**d**) were measured on paws sampled from mice at the end of the imaging session

For the CIA experiments, only three out of eight mice had signs of severe arthritis defined by a score of 4 according to the method of Brand et al. [27]. For the MRI studies, CIA and healthy mice were injected with either USPIO-P258 or USPIO-PEG and the limbs were imaged by MRI at 7 T. Both RARE (raw images in Additional file 2: Figure S9; color overlay in Fig. 3) and FISP (Additional file 2: Figure S10) MRI sequences demonstrated significant negative contrast in the hind limbs of CIA mice, which was not equivalent to any of the negative controls. The negative contrast was evident from the first 12 minutes post injection and persisted until the end of the imaging session (about 2 h) (Fig. 4a-b). The tissue contrasted by USPIO-P258 corresponds to the joints between the tarsal, metatarsal and phalangeal bones and the associated soft tissue. Considering the small size of these bones in mice, it was not possible to distinguish their individual constituents (synovium, cartilage, subchondral bone) by MRI; they appeared as a continuous black trait on the negatively contrasted images of the paw. In addition, the susceptibility effect of iron oxide nanoparticles also contributes to this continuous pattern of contrast. Negative contrast was also observed at the knee.

The ΔSNR% measured on sagittal and coronal RARE images (Fig. 4a-b) confirms the negative contrast produced by USPIO-P258 in hind limbs of CIA mice, where it decreased to about −45 % by 12–25 minutes post injection, and remained constant until 60–70 minutes (around −40 %); at the end of the imaging session (about 2 h), ΔSNR% increased to around −30 %. USPIO-PEG injected into CIA mice produced a decrease to −20 % to −25 % of ΔSNR% 12–25 minutes post contrast, but the negative contrast increased to −13 % to −11 % by the end of the imaging session. The ΔSNR% values measured in healthy mice injected with USPIO-P258 or USPIO-PEG were close to the pre-contrast level and ranged between −5 % and −11 %. These results seem to confirm the RA diagnostic potential of USPIO-P258, probably via specific binding to IL-7Rα.

The biodistribution of contrast agents in the limbs sampled from the mice at the end of the imaging session was evaluated by relaxometry (Fig. 4c-d). The data demonstrated an important accumulation of USPIO-P258 in the limbs of mice with RA as compared to healthy mice (*p* < 0.01). A significant difference (*p* < 0.05) was also observed in comparison with CIA mice injected with USPIO-PEG, confirming the specific targeting of IL-7Rα by USPIO-P258. The results obtained in healthy mice injected with contrast agents were not significantly different from those observed in the control mice not receiving contrast agents.

**Fig. 5:**
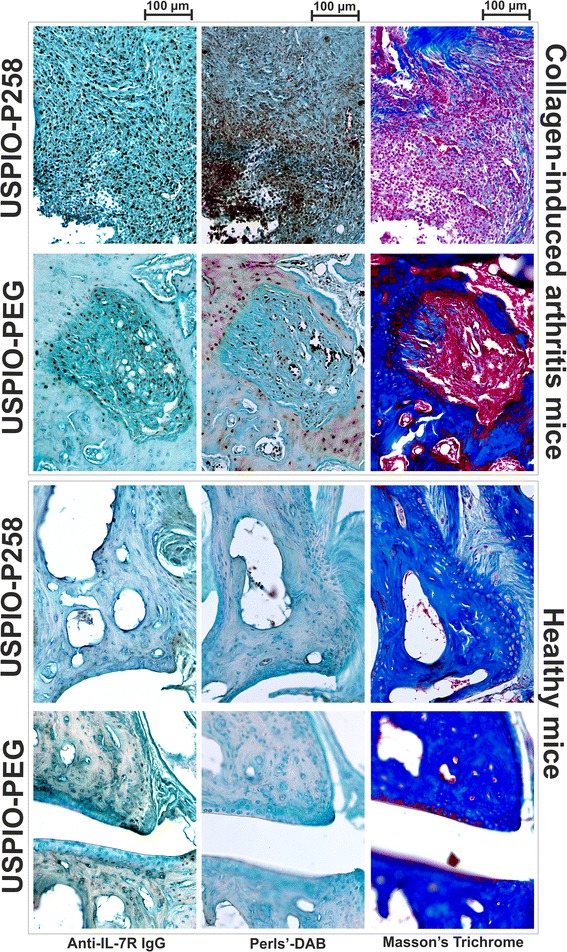
IL-7 receptor (*IL-7Rα*) expression detected by immunohistochemical analysis is compared to ultra-small superparamagnetic particles of iron oxide (*USPIO*)-P258 and USPIO-poly(ethylene glycol) (*USPIO-PEG*) (stained by Perls’-3,3'-diaminobenzidine (*DAB*)) capture in the hind limbs of mice with collagen-induced arthritis studied by magnetic resonance imaging. Histological structures were examined using Masson’s Trichrome staining. For each experimental group, the different staining methods were applied on sequential sections from the same mouse joints

**Fig. 6:**
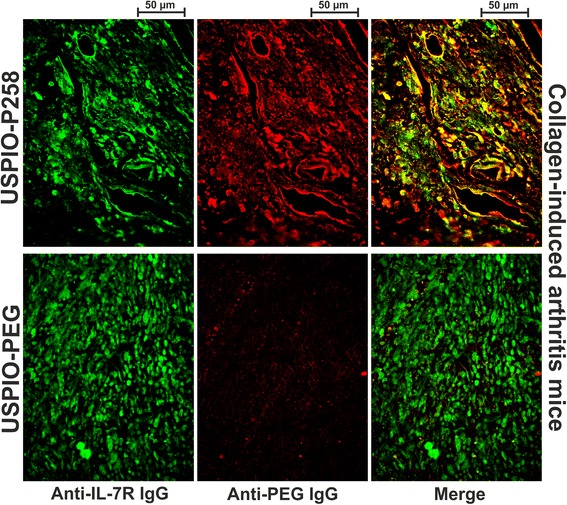
Immunofluorescent co-localization of ultra-small superparamagnetic particles of iron oxide (*USPIO*)-P258 (stained *red* by Texas Red) with IL-7 receptor alpha (*IL-7Rα*) (stained *green* by fluorescein) on the joints of mice with collagen-induced arthritis is evidenced by the *yellow*/*orange* color obtained after merging the microphotographs (*Merge*). No co-localization was observed for USPIO-PEG. Note that anti-PEG antibody recognizes both USPIO-P258 and USPIO-PEG via their PEG coat

The expression of IL-7Rα in the hind limbs of CIA mice subjected to MRI studies was confirmed by immunohistochemical analysis, whereas the Perls’-DAB staining was used to detect contrast agents in the limb tissues (Fig. 5). The connective tissue and different cell types in CIA or healthy tissues were observed using Masson’s Trichrome staining. A prominent leucocyte invasion was observed in the cartilage and synovial tissue of CIA mice compared to healthy mice, which co-localized with high IL-7Rα expression and iron staining when USPIO-P258 was injected. IL-7Rα and USPIO derivatives (USPIO-P258 or USPIO-PEG) were additionally co-localized by immunofluorescence on the same paw section (Fig. 6), USPIO being detected with an anti-PEG antibody. PEG is used both as a stealth coating material and as a linker between P258 and USPIO (Additional file 2: Figure S6A). These results confirm the very good co-localization of IL-7Rα with USPIO-P258 but not with USPIO-PEG, thereby corroborating the specific targeting.

**Fig. 7:**
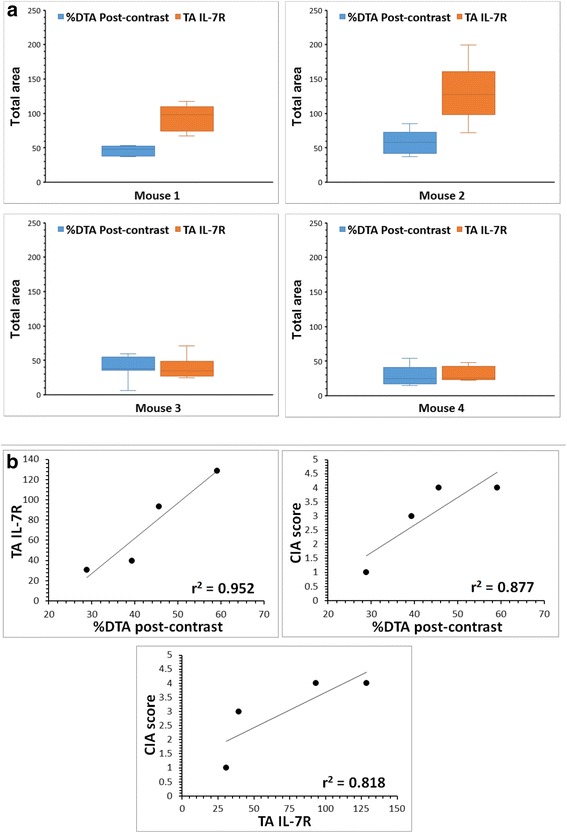
Analysis by ImageJ of the total area (*TA*) occupied by black pixels on post-contrast magnetic resonance (MR) images and by the brown staining of IL-7Rα on immunohistochemical microphotographs. **a** Percentage difference of TA (*%DTA*) on post-contrast MR images as compared to the pre-contrast images and the TA of IL-7 receptor alpha (*IL-7Rα*) staining for each of the four mice in the collagen-induced arthritis (*CIA*) group injected with ultra-small superparamagnetic particles of iron oxide (USPIO)-P258. **b** Correlation between %DTA of post-contrast MR images in the CIA group injected with USPIO-P258, the TA of IL-7R staining and the CIA score

The %DTA in post-contrast images of CIA mice injected with USPIO-P258 and the TA occupied by IL-7Rα staining on the paw sections of the same mice were analyzed using ImageJ software and the results are presented in Fig. 7a. There was strong positive correlation between the mean values of %DTA and those of TA for IL-7Rα staining (*r* ^2^ = 0.952), and between the severity score of CIA and %DTA (*r* ^2^ = 0.877) or TA for IL-7Rα staining (*r* ^2^ = 0.818) (Fig. 7b).

### In vitro characterization of the blocking activity of P725

Jak/STAT is one of the main signaling pathways that are activated by IL-7 upon binding to IL-7Rα [31]. Aiming to validate the ability of P725 to prevent IL-7 binding to its receptor and the triggering of Jak/STAT signaling cascade, a competition experiment was performed by treating ADC-stimulated Jurkat cells with IL-7 and P725; anti-IL-7Rα antibody was used as a positive control. The phosphorylation of Tyr694 in STAT-5 was evaluated by immunofluorescence in treated cells. There was an increase of 424 % increase in phospho-STAT-5 in IL-7-treated cells as compared to ADC-treated controls, which attested for its activation. Anti-IL-7Rα antibody (*p* < 0.01) and P725 (*p* < 0.01) prevented IL-7 binding and STAT-5 activation, demonstrating a pharmacological effect associated with its IL-7 competing activity (Additional file 2: Figures S11A and B).

IL-7R is internalized subsequent to IL-7 binding via clathrin-dependent endocytosis, followed by its recycling or degradation within lysosomes. We therefore repeated the competition experiment with the aim of quantifying the lysosome content of Jurkat cells and thus deduce the outcome of IL-7R when its binding to IL-7 is prevented by P725 (Additional file 2: Figure S12). The effects produced by anti-IL-7Rα antibody and P725 followed the same pattern as that of phospho-STAT-5. IL-7 induced a significant increase in lysosome content (398 % vs. ADC-treated controls), suggesting endocytosis and lysosome degradation of IL-7R. The lysosome content of Jurkat cells significantly diminished after treatment with anti-IL-7Rα antibody (*p* < 0.05) or P725 (*p* < 0.05), suggesting that both competitors prevent IL-7R endocytosis followed by lysosome degradation induced by IL-7. This phenomenon may explain the STAT-5 inactivation produced by both competitors.

## Discussion

The clinical imaging methods currently used for RA diagnosis and monitoring (i.e., radiography, computed tomography, MRI and ultrasound) provide information on bone erosions and joint space narrowing [20, 21]; however, no information is obtained on the cellular and molecular mechanisms of the disease that precede the development of destructive lesions, which sometimes trigger serious debility in less than 2 years in 10 % of cases. Most imaging probes used for RA diagnosis and monitoring are unspecific, although several classic or novel targeted imaging agents have been assessed, such as ^18^F-fluorodeoxyglucose (^18^F-FDG), [^11^C]Choline, (R)-[^11^C]PK11195 and other translocator protein (TSPO)-targeted radiotracers, [^67^Ga]Citrate, [^99m^Tc]- and [^111^In] human immunoglobulin G (HIG), [^99m^Tc]- and [^111^In]anti-E-selectin, [^99m^Tc]- and [^111^In]Octreotide, [^99m^Tc]Anti-TNF-α and [^99m^Tc]Annexin V, etc. [21–23]. Among them, only ^18^F-FDG, TSPO-targeted radiotracers (e.g., ^11^CPBR28, ^18^F-GE-180), the α_v_β_3_-specific imaging probe ^68^Ga-BNOTA-PRGD2 and the PET probe ^18^F-FHBG employed for the imaging of reporter genes, are currently undergoing clinical trials according to the US National Institutes of Health [32]. Examples of new imaging tracers include ^99m^Tc-labeled derivative of octreotide peptide employed to image somatostatin receptor expressed by T lymphocytes [33], and several radiotracers used to monitor therapy response in human or experimental arthritis, such as ^99m^Tc-NTP 15–5 targeted to proteoglycans in the RA joints [34], ^111^In-RGD2 (α_v_β_3_-targeted), ^111^In-anti-fibroblast activation protein antibody and ^111^In-antimurine macrophage antibody [35]. Therefore, there is active research to meet the increasing demand of imaging methods and probes able to provide precocious and reliable information on the clinical outcome, pathophysiological process, the disease severity and location, and the disease response to novel molecular therapies.

The role of IL-7R and IL-7 in the pathogenesis of RA is well-documented. Both molecules are expressed in RA synovial tissue and blockade of the IL-7/IL-7R axis in CIA results in significant clinical improvement. In addition, IL-7Rα was recently identified as a diagnostic marker in early RA, and a marker of severity and poor response to therapy in early and established disease. Our attempts to develop in vivo IL-7Rα imaging tracers are based on these observations, and fit in a novel approach to the taxonomy of inflammatory joint disorders, in which diagnostic and therapeutic decisions are based on the identification of specific molecular pathways, rather than broad clinical diagnostic categories. Thus, we identified several IL-7Rα-specific heptapeptides, which are potential vectors for RA-dedicated imaging probes. Some of them are putative therapeutic agents that work by blocking IL-7 ligation to IL-7R. To the best of our knowledge, no other IL-7Rα-targeted small molecule has been discovered and exploited in the framework of RA diagnosis and treatment.

During the screening of the randomized cyclic heptapeptide phage display library, it was observed that most clones had high affinity against FN type III-like domain of IL-7Rα (A^131^-I^231^), suggesting its involvement in ligand binding. Indeed, specialized literature has shown that 5 of the 12 amino acids (S^51^, F^99^, L^100^, L^101^, I^102^, K^104^, D^122^, H^154^, K^157^, Y^159^, V^160^, H^211^) are involved in IL-7 ligation [36]. As a consequence, excessive preselection against FN has led to a decline in phage pool affinity for IL-7Rα during the third and particularly the fourth round of panning. The peptide sequence of the 12 phage clones selected from the second and the third rounds of panning presented a high frequency of basic (His, Lys) and alcohol (Ser) amino acids, but also a Pro repetition. The first three amino acids are potentially involved in IL-7Rα binding based on the proposed model [36], whereas Pro can induce 20° distortions in the axis of an alpha helix, which suggests that peptides can present a certain 3D conformation. The three best peptide clones (C-PHPQRPA-C, C-KIMKSMP-C and C-ASACPPH-C) characterized by the highest specific affinity against IL-7Rα have shown interesting homologies with molecules involved in signal transduction, cell adhesion, extracellular matrix, cytoskeletal organization, cell migration, embryogenesis and inflammation, proving that their selection was not accidental.

Before synthesis, peptide C-PHPQRPA-C presented the highest binding specificity, whereas peptide C-ASACPPH-C was the weakest candidate. However, after synthesis, peptide C-PHPQRPA-C (encoded as P722) had lost its affinity against IL-7Rα, displaying equivalent binding to FN. Peptide C-KIMKSMP-C (encoded as P725) had better affinity for FN than for IL-7Rα, but its μM K_d_ indicated stronger binding than that of P722. To our surprise, the highest specific binding to IL-7Rα was observed in the case of peptide C-ASACPPH-C (encoded as P726), which thus became the most promising candidate for diagnostic applications. Aiming to eventually improve its affinity constant, the peptide was synthesized in a linear version (encoded as P258), checking in this way the necessity of Cys-constraint. This chemical strategy has indeed enhanced its affinity in the order of nanomolar and improved its specificity against IL-7Rα. The linear peptides present a more flexible spatial conformation, with functional groups optimally exposed for a chemical interaction with the targeted biomarker [37].

One can conclude that peptide P258 presents a chemical interaction with IL-7Rα, the conformational compatibility (imposed by Cys-constraint) not being a prerequisite. Peptide P258 was thus subsequently used to functionalize a MRI contrast agent such as USPIO (USPIO-P258). This reversal of the affinity parameters of the three candidate peptides after their synthesis is explained by the monovalent exposure to the target, after being presented in a pentavalent display on the phage entity. It is very well-known that pentavalent presentation is responsible for an avidity effect that may facilitate the binding of certain peptides to their target.

In our experimental conditions, IL-7 was characterized by a K_d_ (1.7 × 10^−7^ M) close to that of P258, being inferior to the value reported previously (i.e., 2 × 10^−10^ M against the high-affinity IL-7R and 10^−8^ M against the low-affinity IL-7R) [38]. The truncated recombinant IL-7Rα protein (241 amino acids instead of 459 amino acids), removed from its cellular environment, may be responsible for the lower affinity constant observed in our experimental conditions. In addition, two antibodies were used to detect IL-7, which means that additional rinsing steps may contribute to extensive protein removal and lower apparent affinity. However, an interesting observation was that P725 displayed competitive abilities against IL-7, which highlighted its role as a potential therapeutic agent by blocking IL-7 binding to its receptor.

With regard to imaging applications of our IL-7Rα-targeted peptide, USPIO-P258 had good ability to distinguish stimulated from non-stimulated Jurkat cells and its binding to IL-7Rα co-localized with anti-IL-7Rα antibody, confirming its specificity. The blood clearance of USPIO-P258 is much faster than that of USPIO-PEG, and its elimination is likely to mainly occur via a renal pathway. In addition, USPIO-P258 does not seem to accumulate in the main organs, which are simply transited via the blood stream. We have previously observed [24, 25] that peptide grafting to USPIO is responsible for enhanced blood clearance, an enlarged VD_ss_ and increased urinary excretion, probably due to diminished PEG grafting that is partly replaced by peptides on the surface of nanoparticles. Moreover, these biodistribution and pharmacokinetic properties are amplified by peptides with a hydrophilic character.

USPIO-P258 produced a significant negative contrast in the paws of CIA mice, which was not equivalent to any of the control mice. The negative contrast corresponded to the tarsal, metatarsal and phalangeal joints, and persisted for about 2 hours post injection. The contrast agent accumulation in the hind limbs of CIA mice was confirmed by relaxometry and histochemical analysis at the end of the imaging session. The contrast observed at long image acquisition times suggests the specific binding to the targeted receptor, as most of the contrast agent has been cleared from the blood. On histological examination, USPIO-P258 co-localized with IL-7Rα expression in the paws of CIA mice, attesting for its specific accumulation at this level. A non-specific accumulation of USPIO-PEG was also observed in the diseased paws, probably as a result of its phagocytosis by the local macrophages, but its capture was less important as compared to USPIO-P258. Taken together, these results confirm the imaging ability of USPIO-P258 as an IL-7Rα imaging marker, the diagnostic faculty being furthermore confirmed by the high positive correlation between its accumulation in the diseased paws, the IL-7Rα expression and the disease severity.

One of the most important signaling pathways that are activated by IL-7 ligation to its receptor is Jak/STAT, which triggers the phosphorylation of cytoplasmic tyrosine kinases associated with IL-7Rα and the common γ-chain, respectively. Once activated, Jak1 can phosphorylate the Tyr^449^ residue of IL-7Rα, which recruits the transcription factor STAT5 (a heterodimer comprising STAT5a and STAT5b) that is itself tyrosine-phosphorylated by Jak. Phosphorylated STAT5 can dimerize and translocate to the nucleus, where it regulates the transcription of several genes involved in T cell survival and proliferation [2–4]. The inhibition of IL-7 engagement with its receptor by a specific competitor may have potential therapeutic effects in various inflammatory conditions such as RA. The competitive character of P725 was thus confirmed by immunofluorescence, using ADC-stimulated Jurkat cells as a model. P725 inhibited IL-7-induced STAT5 activation by 82 %, which was similar to that produced by anti-IL-7Rα antibody (86 %).

It has been shown that IL-7Rα is rapidly inactivated by lysosome and proteasome-dependent degradation subsequent to its endocytosis triggered by IL-7 activation. In fact, IL-7 signal transduction requires clathrin-dependent endocytosis of IL-7Rα followed by its degradation by proteolysis [30]. In order to check whether STAT5 inactivation induced by P725 treatment is associated with a diminished IL-7Rα endocytosis, we have indirectly measured the lysosome content of Jurkat cells in the same experimental conditions as for STAT5. Our results confirm the increased lysosome content of Jurkat cells stimulated by IL-7, and suggest that P725 ligation to IL-7Rα blocks IL-7 binding and inhibits the endocytosis and lysosome-dependent degradation of IL-7Rα.

## Conclusions

In the present work, we designed IL-7Rα-targeted peptides. The design of cytokine agonists and antagonists of small molecular size is of notable pharmaceutical interest in RA [35] and our work integrates with this general optic. The two IL-7Rα-targeted heptapeptides are hydrophilic and ionized at physiological pH, the blocking one being cyclic (P725), whereas the imaging peptide is linear (P258). The potential blocking effect of P725 was validated in vitro, by preventing STAT5 activation induced by IL-7. Considering its equivalent affinity for IL-7Rα and FN, P725 is not a suitable candidate for molecular imaging applications due to the high potential background generated by the ubiquitously expressed FN. P258 grafted to USPIO produced strong negative contrast in experimental arthritis, even at two hours post-injection when the blood concentration of the imaging probe was very low (18 % from C_0_). The co-localization of USPIO-P258 with IL-7Rα-expressing cells in CIA synovitis demonstrates its specific binding to the targeted receptor. In CIA conditions, USPIO-P258 is able to discriminate the level of IL-7R expression and the disease severity.

## Additional files

Additional file 1: Methods. (PDF 370 kb)
Additional file 2: Figures. (PDF 3 mb)

## Abbreviations

ABTS: 2,2´-Azino-bis(3-Ethylbenzothiazoline-6-sulfonic acid)
ADC: 5-Aza-2′-deoxycytidine
BLAST: basic local alignment search tool
BSA: bovine serum albumin
b.w.: body weight
CIA: collagen-induced arthritis
Cl_tot_: total clearance
DAB: 3,3'-diaminobenzidine
DAPI: 4',6-diamidino-2-phenylindole
DTA: difference in total area
ELISA: enzyme-linked immunosorbent assay
ERK: extracellular signal-related kinase
FISP: fast imaging with steady-state precession
FN: fibronectin
HRP: horseradish peroxidase
IC_50_: half maximal inhibitory concentration
IL-7Rα: interleukin-7 receptor alpha
Jak1: Janus kinase 1
K*_d_: apparent dissociation constant
MAPK: mitogen activated protein kinase
MRI: magnetic resonance imaging
MSME: multi-slice-multi-echo
NS: non-stimulated
OD: optical density
PI3K: phosphatidylinositol 3-kinase
PBS: phosphate-buffered saline
PBST: phosphate-buffered saline supplemented with Tween-20
PCS: photon correlation spectroscopy
PEG: poly(ethylene glycol)
PFBB: protein-free blocking buffer
pI: isoelectric point
r_2_: transverse relaxivity
R_2_: transverse relaxation rate
RA: rheumatoid arthritis
RANK: receptor activator of nuclear factor-κB
RANKL: receptor activator of nuclear factor-κB ligand
RARE: rapid acquisition with relaxation enhancement
ROIs: regions of interest
RRFL: relative ratio of fluorescent labeling
SD: standard deviation
SI: signal intensity
SNR: signal-to-noise ratio
STAT5: signal transducer and activator of transcription 5
T_2_: transverse relaxation time
TA: total area
T_e1/2_: elimination half-life
TGF-β: transforming growth factor-β
TLR: toll-like receptor
TNFα: tumor necrosis factor-alpha
USPIO: ultra-small superparamagnetic particles of iron oxide
VDss: volume of distribution steady state
