# Supplementary material for: Screening for peptides targeted to IL-7Rα for molecular imaging of rheumatoid arthritis synovium
Source: Arthritis Res Ther. 2016 Oct 12;18:230. doi: 10.1186/s13075-016-1133-8 (PMC5059943; doi:10.1186/s13075-016-1133-8)
Supplement: Additional file 1: — Methods. (PDF 370 kb) [file 13075_2016_1133_MOESM1_ESM.pdf]

Additional File 1: Methods

Screening for peptides targeted to IL-7Rα for molecular imaging of rheumatoid arthritis synovium

Carmen Burtea[1], Sophie Laurent[1,5], Tuba Sanli[1], Deborah Fanfone[1], Aude Devalckeneer[1], Sébastien Sauvage[2,3,7], Marie-Claire Beckers[4,8], Sandrine Rorive[2,3], Isabelle Salmon[2,3], Luce Vander Elst[1,5], Bernard R. Lauwerys[6], Robert N. Muller[1,5]

[1] Department of General, Organic and Biomedical Chemistry, NMR and Molecular Imaging Laboratory, University of Mons, Avenue Maistriau 19, Mendeleev Building, B-7000 Mons, Belgium; [2] Department of Pathology, Erasme Hospital, Université Libre de Bruxelles, Route de Lennik 808, 1070 Brussels, Belgium; [3] DIAPath, Center for Microscopy and Molecular Imaging, 8, rue Adrienne Bolland - 6041 Gosselies, Belgium; [4] Eurogentec S.A. Liège Science Park, Rue du Bois Saint-Jean 5, B-4102 Seraing, Belgium; [5] Center for Microscopy and Molecular Imaging, 8, rue Adrienne Bolland - 6041 Gosselies, Belgium; [6] Pôle de pathologies rhumatismales inflammatoires et systémiques, Institut de Recherche Expérimentale et Clinique, Université Catholique de Louvain, Avenue Mounier 53, 1200 Brussels, Belgium; [7] Present address: Molecular Pathology Laboratory, ONCODNA – The Cancer Theranostic Company, 25 Av. Georges Lemaître, 6041 Gosselies, Belgium; [8] Present address: ASIT Biotech s.a., 3 Rue des Chasseurs Ardennais, 4031 Angleur, Belgium

Contents

1. The experiment of phage display and characterization of the candidate phage clones...... 2
1.1. Phage display library and E. coli host strain 2
1.2. The panning of phage display library against IL-7Rα...... 2
1.3. Evaluation of the phage clones' affinity for IL-7Rα 3
2. Characterization of the selected IL-7Rα-binding peptides...... 4
2.1. Estimation of K_d 4
2.2. Estimation of IC_{50}...... 4
2.3. Binding of peptides to Jurkat cells and co-localization with IL-7Rα by immunofluorescence 5
2.4. Immunohistochemistry detection of biotinylated P258 and of IL-7Rα on human knee of RA patients...... 5
2.5. Phospho-STAT5 detection on Jurkat cells and modulation by peptide P725 6
2.6. Evaluation of the lysosome content of Jurkat cells 7
3. Characterization of the imaging probe 7
3.1. Evaluation of USPIO-P258 binding to Jurkat cells...... 7
3.2. In vivo evaluation of USPIO-P258: pharmacokinetics, biodistribution and molecular imaging...... 8
3.3. IL-7Rα expression, Perls'-DAB staining of USPIO derivatives on paw samples and Masson's Trichrome staining; immunofluorescent co-localization of IL-7Rα with USPIO-P258...... 9
References 10

1. The experiment of phage display and characterization of the candidate phage clones

1.1. Phage display library and E. coli host strain

The Ph.D.-C7C™ phage display library (New England BioLabs® Inc., Westburg b.v., Leusden, The Netherlands) is based on a disulfide-constrained combinatorial library of random heptapeptides fused to the minor coat protein (pIII) of M13 phage. This phage library is derived from the common cloning vector M13mp19, which carries the lacZα gene. For this reason, the phage plaques appear blue when plated on media containing Xgal (5-Bromo-4-chloro-3-indolyl-β-D-galactopyranoside, Sigma-Aldrich, Bornem, Belgium) and IPTG (isopropyl β-D-thiogalactopyranoside, ICN Biomedicals Inc).

The *E. coli* host ER2738 (E. coli K12 ER2738, New England BioLabs®) is an F^+, tetracycline-resistant strain.

1.2. The panning of phage display library against IL-7Rα

The display library was panned against recombinant human IL-7 Rα/Fc chimera (R&D Systems, Abington, UK), which was immobilized alternatively on Dynabeads® Protein A or G (Invitrogen Dynal, Merelbeke, Belgium) during the four rounds of biopanning. To increase the peptide specificity and stringency, the following strategy was used during the panning protocol: (1) target concentration bound to Dynabeads was of 100 nM (the 1st and the 2nd rounds) and 75 nM (the 3rd and the 4th rounds); (2) the immobilization system (Dynabeads Protein A / Dynabeads Protein G) was alternated at each round of panning; (3) the incubation time with the target was reduced stepwise (i.e., 60 min for rounds 1 and 2; 45 min for round 3; 30 min for round 4); (4) the incubation times with background molecules [BSA blocked Dynabeads; Fc-IgG (human IgG, Fc fragment from plasma, Calbiochem, VWR, Leuven, Belgium) immobilized to Dynabeads; fragment 3 of recombinant human fibronectin-1 (R&D Systems) immobilized to Dynabeads] were increased stepwise (i.e. 15 min for rounds 1 and 2, 20 min for round 3, 25 min for round 4); (5) the Dynabeads were blocked (1h, 4°C) with a blocking buffer (5mg/mL of BSA in PBS, pH 7.4); (6) the Tween-20 concentration was increased stepwise (i.e., 0.1% - 0.5%) in the incubation and rinsing buffer, which was sterile PBS (per L: 8g NaCl, 0.2 g KCl, 2.31 g Na_2HPO_4x12 H_2O, 0.2 g KH_2PO_4, pH 7.4).

After blocking the nude or coated Dynabeads, they were washed 4 times with 0.2 mL of PBS completed with Tween-20 (PBS-T), and finally were suspended in 0.19 mL of sterile PBS-T by taking care to bring the immobilized protein concentration to 100 or 75 nM. The

phage library (2x10^{11}) was afterward added (0.01 mL of the original phage display library, or variable volume for the next rounds of panning) to the Dynabeads free of protein but blocked with BSA, and incubated at room temperature on the roller. The fluid was then removed, and thereafter was incubated with Fc/IgG-coated Dynabeads (room temperature, roller device). The Dynabeads were removed each time by using a magnet. The fluid was then transferred to a fibronectin coated well (FN, 100 µg/mL in 0.1 M $NaHCO_3$, pH 8.6, immobilized overnight at 4°C) of an ELISA plate (the well was blocked for 1h at 4°C with 0.3 mL/well of blocking buffer containing 0.1 M $NaHCO_3$, 0.02% NaN_3, 0.5% BSA) and incubated at room temperature. The solution was thereafter incubated (room temperature, roller device) with IL-7Rα-coated Dynabeads. The fluid was subsequently removed, and Dynabeads were washed 8 times with PBS-T and 2 times with PBS. The bound phages were then eluted (10 min, room temperature, roller device) by suspending Dynabeads in 0.2 mL of sterile 0.2 M Glycine-HCl, pH 2.2, completed with 1 mg/mL BSA. The eluted phage solution was immediately neutralized with 0.03 mL of 1 M Tris-HCl, pH 9.1, while the phage titer was evaluated by infecting *E. coli* cultures. The phage pool was amplified after *E. coli* infection with the aim to be used as an input for the next round of panning.

1.3. Evaluation of the phage clones' affinity for IL-7Rα

The pools of the four rounds of panning and the 102 phage clones isolated from the 2nd and the 3rd rounds of panning were screened against IL-7Rα diluted at a concentration of 10 µg/mL in 0.1 M $NaHCO_3$, pH 8.6, and immobilized overnight at 4°C. The control wells were coated with 5 mg/mL of BSA or with 10 µg/mL of FN and used to evaluate the non-specific binding of the phages. The phages were diluted at a concentration of 2.5x10^{11} virions/mL (for the phage pools) or of 10^{12} virions/mL (for individual phage clones) in PBS supplemented with 0.5% Tween-20 (PBST), and were incubated with both test and control wells (2h, 37°C). The bound phages were detected with HRP-conjugated anti-M13 antibody (Amersham Pharmacia Biotech Benelux, Roosendaal, The Netherlands) diluted 1:5000 in THBS completed with 5 mg BSA/mL. The plate was washed with PBST after each incubation step (microplate washer Adil Instruments LP21, Fisher-Bioblock, Tournai, Belgium). The staining reaction was developed with ABTS [2,2´-Azino-bis(3-Ethylbenz-thiazoline-6-sulfonic acid), diamonium salt (Sigma-Aldrich, Bornem, Belgium)] solution (22 mg ABTS in 100 mL of 50 mM sodium citrate, pH 4.0) completed with 0.05% H_2O_2. The OD_{405} was measured using a microplate reader (StatFax-2100, Awarness Technology, Fisher Bioblock Scientific).

To estimate the apparent dissociation constant (K^*_d), the selected phage clones were incubated with IL-7Rα or with FN at a concentration that ranged from $2x10^{13}$ to $3.91x10^{10}$ virions/mL. All the other protocol steps are identical to those described above for the global screening of the phage clones. The specificity of binding was confirmed for 3 of the selected clones by pre-incubating the immobilized IL-7Rα with a range of human recombinant IL-7 (R&D Systems) concentrations ($1.83x10^{-5}$ M – $1.04x10^{-10}$ M). After 30 min of incubation, the phages were added at a concentration equal to their K^*_d and the incubation was continued for one more hour. The phages bound to the target were detected with HRP-conjugated anti-M13 antibody as described above.

2. Characterization of the selected IL-7Rα-binding peptides

2.1. Estimation of K_d

IL-7Rα and FN were immobilized as described above and the wells were blocked with PFBB (Protein-Free Blocking Buffer, Perbioscience, Erembodegem, Belgium) 1h at room temperature. Serial dilutions of biotinylated peptides (10^{-3} – 10^{-6} M or 10^{-5} – 10^{-8} M) were prepared in PBS and 0.1 mL of each peptide dilution was transferred to coated wells, followed by an incubation of 2h at 37°C. The wells were subsequently incubated (1h, room temperature) with 1 µg/mL of a goat anti-biotin antibody (Vector Labconsult, Brussels, Belgium) diluted in phosphate buffer (10 mM phosphate, 0.15 M NaCl, pH 7.8) supplemented with 0.5% bovine serum albumin (BSA), followed by 0.2 µg/mL of a peroxidase conjugated anti-goat antibody made in horse (Vector Labconsult) diluted in phosphate buffer supplemented with 0.1% Tween-20 and 0.5% BSA (1h, room temperature). After each incubation step, the wells were washed with PBST-0.05% using a microplate washer. The staining reaction was developed with 0.1 mL/well of ABTS/H_2O_2, and OD_{405} was measured with a microplate reader.

2.2. Estimation of IC_{50}

IL-7Rα was immobilized on ELISA plates and the wells were blocked as described at point 2.1. The K^*_d of IL-7 was determined by aiming to be compared with that of peptides, but also to use it during the competition experiments with peptides. For this purpose, a range of IL-7 concentrations ($7x10^{-7}$ M – $2.14x10^{-11}$ M) were incubated (37°C, 2h) with immobilized IL-7Rα. The bound IL-7 was then detected with 5 µg/mL of a polyclonal goat anti-human IL-7 antibody (R&D Systems) followed by 5 µg/mL of a horse anti-goat antibody coupled to HRP (Vector Labconsult).

To estimate the IC_{50} of the 3 peptides, they were pre-incubated (37°C, 30 min) with IL-7Rα coated wells in a range of concentrations (10^{-3} M – 10^{-7} M). IL-7 was then added at a concentration equal to its own K^*_d and the incubation continued for another 90 min. IL-7 bound to the target was detected as described above.

2.3. Binding of peptides to Jurkat cells and co-localization with IL-7Rα by immunofluorescence

Jurkat cells were cultured (37°C in a humidified 5% CO_2 incubator) at a concentration of less than 10^6 cells/mL in RPMI-1640 culture medium supplemented with 10% newborn calf serum heat inactivated and 1% antibiotic-antimycotic (all from Invitrogen). The cells were stimulated during 4 days with 0.4 µM of 5-Aza-2′-deoxycytidine (ADC, Sigma-Aldrich) [1]. The stimulated cells were immobilized on coverslips pre-coated with 0.01% poly-L-lysine (Sigma-Aldrich) and fixed (15 min, room temperature) in a solution of 4% formaldehyde (Sigma-Aldrich) diluted in PBS. After blocking the cells with PFBB, they were co-incubated (overnight, 4°C) with 10 µg/mL of a mouse anti-human IL-7Rα monoclonal antibody (Sigma-Aldrich) and with biotinylated peptides P258 (0.04 µM) or P725 (5 µM). Subsequently, the cells were incubated (1h, room temperature) with 10 µg/mL of a goat anti-biotin antibody (Vector Labconsult) diluted in phosphate buffer. They were then co-incubated (1h, room temperature) with 20 µg/mL of fluorescein-conjugated rabbit anti-goat antibody and with 20 µg/mL of Texas Red horse anti-mouse antibody (both from Vector Labconsult). The coverslips were finally mounted on slides with Vectashield mounting medium for fluorescence with DAPI (Vector Labconsult) and observed on a DM2000 Leica microscope equipped with a DFC 425C camera (Leica Microsystems, Groot Bijgaarden, Belgium).

2.4. Immunohistochemistry detection of biotinylated P258 and of IL-7Rα on human knee of RA patients

After dewaxing the knee sections by classical procedures, the epitopes were unmasked by treatment with citrate solution, whereas the endogenous peroxidases were blocked with 0.3% H_2O_2 in PBS and the non-specific epitopes were blocked with PFBB.

For IL-7Rα detection, the sections were incubated (4°C, overnight) with 10 µg/mL of mouse anti-human IL-7Rα monoclonal antibody (Sigma-Aldrich), followed by 1h incubation at room temperature with a peroxidase-conjugated monoclonal anti-mouse antibody produced in goat (Sigma-Aldrich) diluted 200 times in TBS (50mM Tris/HCl, 150mM NaCl, pH 7,5). The sections were subsequently incubated for 5 min with 50 mM Tris-HCl, pH 7.4 and then

stained with 0.05% 3,3'-diaminobenzidine (DAB) completed with 0.02% H_2O_2 in PBS. Finally, they were counterstained with Hemalun and Luxol fast blue and mounted in a permanent medium.

To validate the binding of biotinylated P258 to human knee sections, the endogenous biotin was first blocked with a blocking kit (Invitrogen). The histologic sections were then incubated (4°C, overnight) with 1 µM of P258, followed by an incubation (1h, room temperature) with 5 µg/mL of a goat anti-biotin antibody (Vector Labconsult). Finally, the slices were incubated (1h, room temperature) with 1 µg/mL of a peroxidase conjugated anti-goat antibody made in horse (Vector Labconsult). The staining and counterstaining were performed as described above for anti-IL-7Rα monoclonal antibody.

2.5. Phospho-STAT5 detection on Jurkat cells and modulation by peptide P725

Jurkat cells were cultured and stimulated with ADC as described above. The experiment was performed in triplicate on stimulated and non-stimulated cells that were distributed in several culture tubes at a concentration of 10^6 cells/mL and treated (37°C in a humidified 5% CO_2 incubator) with different compounds diluted in the culture medium as follows: (A) cells treated for 48h with 6 ng/mL of IL-7 (eBioscience, Vienne, Austria); (B) cells pre-incubated for 30 min with 1 mM of P725; (C) cells pre-incubated for 30 min with 10 µg/mL of mouse anti-human IL-7Rα monoclonal antibody (Sigma-Aldrich). In the case of samples B and C, the pre-incubation was followed by 48h of incubation with 6 ng/mL of IL-7. The solutions were replaced every day after centrifuging the culture tubes and removing the precedent solutions by pipetting.

At the end of the treatment period, the cells were immobilized on poly-L-lysine coated coverslips and fixed with 4% formaldehyde as described above. After three times rinsing with PBS, the cells were treated with 100% methanol for 10 min at -20°C. They were then rinsed once with PBS and blocked (1h, room temperature) with a solution of 5% normal goat serum and 0.3% Triton X-100 prepared in PBS. Subsequently, the cells were co-incubated (4°C, overnight) with human Phospho-Stat5 (Tyr694 D47E7 XP®) antibody made in rabbit diluted 100 times and anti-Pan-keratin (C11) antibody made in mouse diluted 400 times (both from Bioké, Leiden, The Netherlands), both prepared in a solution of PBS containing 1% BSA and 0.3% Triton X-100. The primary antibodies bound to the cells were then detected by a co-incubation (1h, room temperature) with 20 µg/mL of a fluorescein-conjugated goat anti-rabbit antibody and 20 µg/mL of a horse Texas Red-conjugated anti-mouse antibody (both from

Vector Labconsult), both diluted in phosphate buffer. The coverslips were finally mounted on slides with Vectashield mounting medium for fluorescence with DAPI (Vector Labconsult).

2.6. Evaluation of the lysosome content of Jurkat cells

Jurkat cells were stimulated with ADC and treated with IL-7, peptide P725 and anti-IL-7Rα antibody as described at point 2.5. For lysosome tracking, cells were incubated (37°C in a humidified 5% CO_2 incubator) for 5 min with a solution of 2 µg/mL of Hoechst 33342, followed by an incubation of 1 min in a solution of 100 nM of Lysotracker® Red DND-99 (Image-mITT LIVE lysosomal and nuclear labelling kit, Life technologies, Merelbeke, Belgium), both diluted in HBSS (140 mg $CaCl_2$, 100 mg $MgCl_2{\cdot}6H_2O$, 100 mg $MgSO_4{\cdot}7H_2O$, 400 mg KCl, 60 mg KH_2PO_4, 350mg $NaHCO_3$, 8 g NaCl, 48mg Na_2HPO_4, pH 7,4). The cells were then mounted between microscope slides and coverslips, observed at microscope and the RRFL was evaluated as described above at point 2.5.

3. Characterization of the imaging probe

3.1. Evaluation of USPIO-P258 binding to Jurkat cells

Non-stimulated Jurkat cells or stimulated with ADC as described above were incubated ($4x10^6$ cells in 2 mL in culture medium; n=3/experimental group; room temperature, mechanical agitation) with USPIO-P258 or USPIO-PEG at an iron concentration of 1-3 mM. The cells were subsequently rinsed three times with PBS and the solution was removed by centrifugation (4500 rpm, 10 min). After mineralization (200 µL of 5N HCl for $2x10^6$ cells, 3h at 80°C, Bain Marie), the iron concentration in cell samples was determined by relaxometry on a Bruker Minispec Mq60 (Karlsruhe, Germany) based on a calibration curve. For MRI analysis and the measurement of the transverse relaxation time (T_2), the cells ($2x10^6$) incubated with contrast agents were suspended in 200 µL of 2% gelatin prepared in PBS. The MRI acquisitions were performed at 15°C on a 300 MHz (7T) Bruker Biospec imaging system (Ettlingen, Germany). A T_2-weighted RARE (Rapid Acquisition with Relaxation Enhancement) sequence (TR = 4000 ms, effective TE = 200 or 300 ms, RARE factor = 4, NEX = 4, FOV = 3 cm x 3 cm, matrix = 256x256, slice thickness = 1 mm, spatial resolution = 117 µm) was used for cells visualization. The T_2 was measured with an MSME (Multi-Slice-Multi-Echo) sequence (TR = 5462 ms, TE = 80 ms, 16 echoes, FOV = 4.42 cm x 4.47 cm, matrix = 256x256, slice thickness = 1 mm, spatial resolution = 173x175 µm). The results were expressed as transverse relaxation rates ($R_2 = 1/T_2$) normalized to gelatine (R_2^{Norm}) and measured in s^{-1}.

USPIO-P258 was co-localized with IL-7Rα expressed by Jurkat cells using an immunofluorescence protocol. At the end of the MRI studies, the cells were recovered from gelatine by melting it at 37°C, followed by cell sedimentation by centrifugation for 30 min at 4500 rpm and 37°C. The cells were then resuspended in 50 µL of PBS and immobilized on poly-L-lysine coated coverslips, fixed and blocked as described above at point 2.3. The cells were then co-incubated overnight at 4°C with 10 µg/mL of a mouse anti-human IL-7Rα monoclonal antibody (Sigma-Aldrich) and with 2 µg/mL of rabbit monoclonal anti-PEG antibody (Bio-Connect / Epitomics, Huissen, The Netherlands), both diluted in a solution of PBS containing 2 mg/mL of BSA. After rinsing the cells three times with PBS, they were subsequently co-incubated (1h, room temperature) with 20 µg/mL of Texas Red horse anti-mouse antibody and 20 µg/mL of fluorescein goat anti-rabbit antibody (both from Vector Labconsult) diluted in phosphate buffer. The coverslips were finally mounted on slides with a mounting medium for fluorescence (Vector Labconsult). The RRFL was evaluated as described above at point 2.5.

3.2. In vivo evaluation of USPIO-P258: pharmacokinetics, biodistribution and molecular imaging

The experiments fulfil the requirements of the Ethical Committee of our institution.

For pharmacokinetics and biodistribution evaluation, NMRI mice (n=3/experimental group; average weight 30 g; Harlan, Horst, The Netherlands) were anesthetized with 60 mg/kg b.w., i.p., of Nembutal (Sanofi, Bruxelles, Belgium) and were injected with 100 µmol Fe/kg b.w. of USPIO-P258. The negative control animals were left untreated. The blood, urine and organs (kidneys, liver, spleen and lungs) were collected at various time intervals (1, 2, 2.5, 5, 15, 30, 45, 65, 90 and 120 minutes) after the injection of the contrast agent. The blood was collected on heparin for plasma isolation. The organs were rinsed with 15 ml of PBS by transcardial perfusion, and they were sampled for the R_2 measurement on a Bruker Minispec mq60 working at 60 MHz and 37°C. The concentration of USPIO derivatives in blood plasma and urine was calculated by relating the R_2^{Norm} (normalized by subtracting the R_2 of negative control animals) of test samples to the transverse relaxivity, r_2, of the contrast agent as measured in blood plasma and urine. A two-compartment distribution model was used to calculate the pharmacokinetic parameters such as the elimination half-life ($T_{e1/2}$), the volume of distribution steady state (VD_{ss}) and the total clearance (Cl_{tot}).

For molecular imaging studies, autoimmune rheumatoid arthritis (RA) was induced in DBA/1 male mice (n = 4 per experimental group) aged 10 weeks (Harlan) by immunization

with an emulsion of complete Freund's adjuvant and type II collagen from chicken sternal cartilage (Sigma-Aldrich) as described by literature [1]; the healthy control mice were left untreated (n = 4 per experimental group). Collagen was prepared in 10 mM acetic acid at a concentration of 4 mg/mL. Complete Freund adjuvant (CFA, Sigma-Aldrich) containing 1 mg/mL of heat-killed *Mycobacterium tuberculosis* was used to prepare an injectable emulsion, which was complemented with 3 mg/mL of *M. tuberculosis* (Voigt Global Distribution Inc, Lawrence, USA) to attain a final concentration of 4 mg/mL. The collagen solution was mixed with complemented CFA (1:1) by using an IKA-Werke homogenizer. Mice were anesthetized with 30 mg/kg of Nembutal and 50 µL of the emulsion containing 100 µg of collagen were injected intradermally in the tail; an equivalent dose was re-injected 3 weeks later. The mice were used for MRI, biodistribution and (immuno)histochemistry studies 18-21 days after the second injection.

The mice were distributed into 4 groups of 4 mice each (2 RA and 2 healthy groups) and were injected i.v. in the caudal vein at a dose of 100 µmol Fe/kg b.w. with either USPIO-P258 or USPIO-PEG for MRI studies; the mice not injected with contrast agents were used as controls for relaxometric studies. The acquisition of images started immediately after contrast agent injection with RARE (Rapid Acquisition with Relaxation Enhancement) (TR/TE = 3000/48.7 ms, effective echo = 97.4 ms, RARE factor = 4, NEX = 4, matrix = 256x256, FOV = 4 x 2.3 cm, slice thickness = 1.1 mm, 20 sagittal or coronal slices, spatial resolution = 156x90 µm) or 3D FISP (Fast Imaging with Steady-state Precession) (TR/TE = 18/4 ms, flip angle = 15°, NEX = 4, matrix = 512x256x128, FOV = 3.2x3x3.2 cm, slice thickness = 0.23 mm, spatial resolution = 59x117x234 µm) sequences.

During MRI studies, the mice were anesthetized with 2% isoflurane (Tem Sega, Lormont, France) delivered by airflow through a nose adapter at 70 ml/min. A small-animal monitoring and gating system was used to monitor animal respiration rate. During the acquisition of MR images, the mouse body temperature was maintained at 36–37°C using a warm water circulating system.

3.3. IL-7Rα expression, Perls'-DAB staining of USPIO derivatives on paw samples and Masson's Trichrome staining; immunofluorescent co-localization of IL-7Rα with USPIO-P258

For (immuno)histochemistry studies, paws were fixed in buffered PAF and decalcified in Biodec-R (Bio-optica Milano s.p.a) for 8 days before paraffin embedding. Sections of 5 µm were cut and IL-7Rα expression was evaluated by immunohistochemistry. After dewaxing paw

slices by classical procedures, the endogenous peroxidases were blocked with 0.7% H_2O_2 in PBS, while non-specific epitopes were blocked with 1% BSA in TBS (50 mM Tris/HCl, 150 mM NaCl, pH 7.5). IL-7Rα was detected with mouse anti-human IL-7Rα monoclonal antibody and peroxidase-conjugated monoclonal anti-mouse antibody produced in goat (both from Sigma-Aldrich) as described above at point 2.4.

USPIO derivatives were detected on paw sections by histochemistry, using the Perls'-DAB iron staining protocol. After dewaxing and rehydration, endogenous peroxidases were blocked 15 min with 1% H_2O_2 in PBS. The slices were then incubated for 30 min with Perls' working solution (mixing equal volumes of 5% potassium ferrocyanide and 5% HCl). After rinsing three times for 10 min in distilled water, the tissue was treated for 10 min with 0.05% DAB in PBS, pH 7.4, followed by immersion for 10 min in 0.05% DAB supplemented with 0.033% H_2O_2 prepared in PBS, pH 7.4. After rinsing again three times in distilled water, the sections were counterstained with hemalun and Luxol fast blue and mounted in a permanent medium.

After dewaxing and rehydration, paw sections were blocked with 1% BSA in PBS. USPIO derivatives were then co-localized with IL-7Rα expressing cells by co-incubating (overnight, 4°C) paw sections with 8 µg/mL of rat anti-PEG (Abcam) and 10 µg/mL of mouse anti-IL-7Rα (Sigma-Aldrich) monoclonal antibodies, followed (1h, room temperature, obscurity) by 20 µg/mL of Texas red-conjugated goat anti-rat and 20 µg/mL of Fluorescein-conjugated horse anti-mouse antibodies (both from Vector Labconsults) prepared in phosphate buffer pH 7.8 containing 0.5% BSA and 0.05% Tween-20; sections were then observed under a fluorescence microscope.

The paws' morphology was studied after Masson's trichrome staining of 5 µm sections using the Accustain® kit (Sigma-Aldrich) according to the manufacturer's instructions. Briefly, histological sections were treated overnight with Bouin's solution to strengthen the final coloration. Weigert's iron hematoxylin was employed for nuclei staining, while cytoplasm and muscle were stained with Beibrich scarlet-acid fuchsine. After treating paw sections with phosphotungstic and phosphomolybdic acid, the collagen was stained with aniline blue. Tissue sections were then rinsed in acetic acid and distilled water and finally mounted in a permanent medium after dehydration.

References

1. Kim HR, Hwang KA, Kim KC and Kang I. Down-regulation of IL-7R expression in human T cells via DNA methylation. J Immunol 2007;178:5473-9.
