# Supplementary material for: Screening for peptides targeted to IL-7Rα for molecular imaging of rheumatoid arthritis synovium
Source: Arthritis Res Ther. 2016 Oct 12;18:230. doi: 10.1186/s13075-016-1133-8 (PMC5059943; doi:10.1186/s13075-016-1133-8)
Supplement: Additional file 2: — Figures. (PDF 3 mb) [file 13075_2016_1133_MOESM2_ESM.pdf]

Additional File 2: Figures

Screening for peptides targeted to IL-7Rα for molecular imaging of rheumatoid arthritis synovium

Carmen Burtea[1], Sophie Laurent[1,5], Tuba Sanli[1], Deborah Fanfone[1], Aude Devalckeneer[1], Sébastien Sauvage[2,3,7], Marie-Claire Beckers[4,8], Sandrine Rorive[2,3], Isabelle Salmon[2,3], Luce Vander Elst[1,5], Bernard R. Lauwerys[6], Robert N. Muller[1,5]

[1] Department of General, Organic and Biomedical Chemistry, NMR and Molecular Imaging Laboratory, University of Mons, Avenue Maistriau 19, Mendeleev Building, B-7000 Mons, Belgium; [2] Department of Pathology, Erasme Hospital, Université Libre de Bruxelles, Route de Lennik 808, 1070 Brussels, Belgium; [3] DIAPath, Center for Microscopy and Molecular Imaging, 8, rue Adrienne Bolland - 6041 Gosselies, Belgium; [4] Eurogentec S.A. Liège Science Park, Rue du Bois Saint-Jean 5, B-4102 Seraing, Belgium; [5] Center for Microscopy and Molecular Imaging, 8, rue Adrienne Bolland - 6041 Gosselies, Belgium; [6] Pôle de pathologies rhumatismales inflammatoires et systémiques, Institut de Recherche Expérimentale et Clinique, Université Catholique de Louvain, Avenue Mounier 53, 1200 Brussels, Belgium; [7] Present address: Molecular Pathology Laboratory, ONCODNA – The Cancer Theranostic Company, 25 Av. Georges Lemaître, 6041 Gosselies, Belgium; [8] Present address: ASIT Biotech s.a., 3 Rue des Chasseurs Ardennais, 4031 Angleur, Belgium

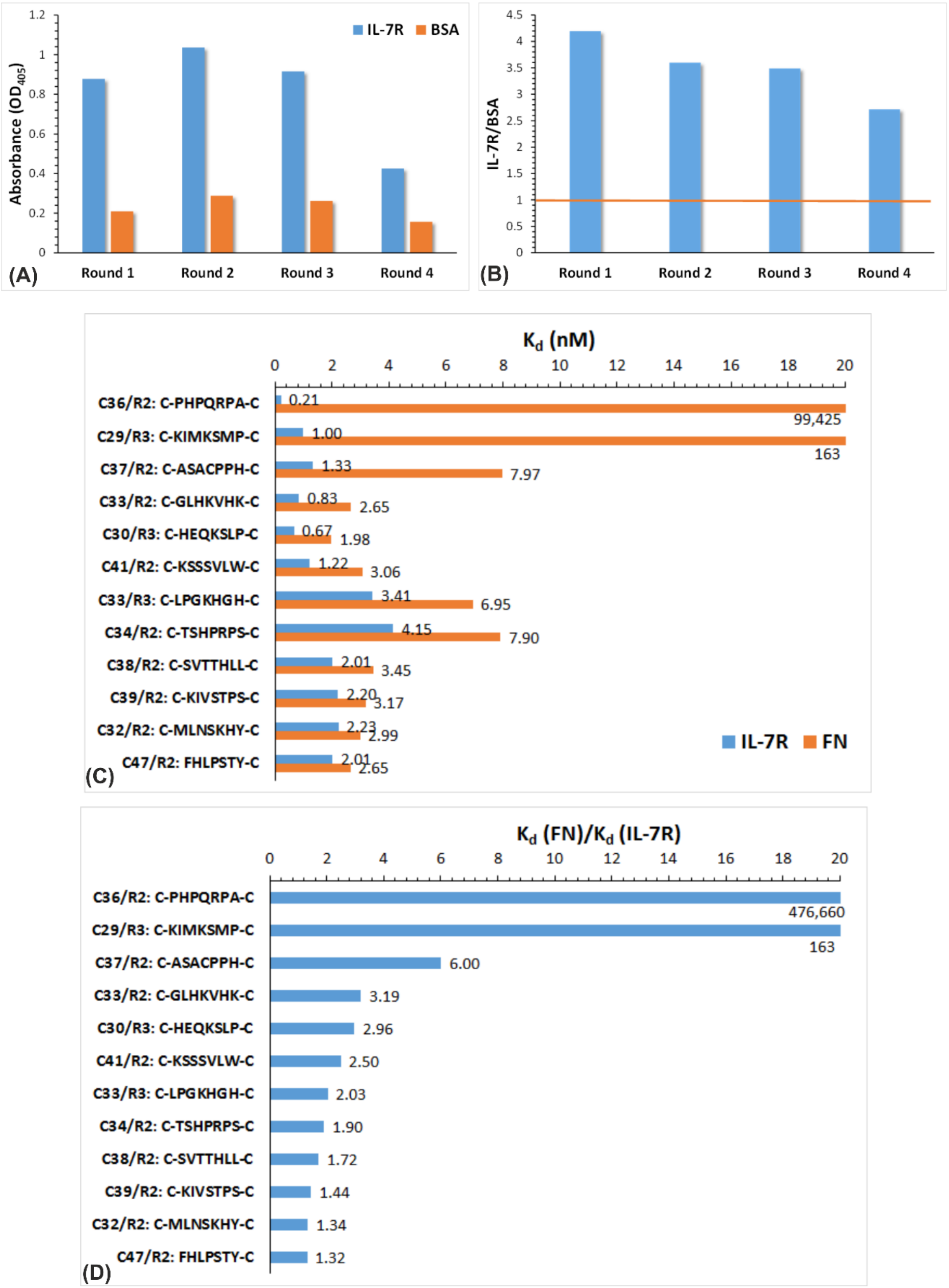

Figure S1. The affinity for IL-7R and control proteins (BSA and FN) of the outputs derived from the four rounds of panning (**A** and **B**) and of the selected phage clones (**C** and **D**) isolated from the pools of the second (R2) and the third (R3) rounds of panning. The results are expressed as absolute values (**A** and **C**) or as a ratio between IL-7R and control proteins (**B** and **D**).

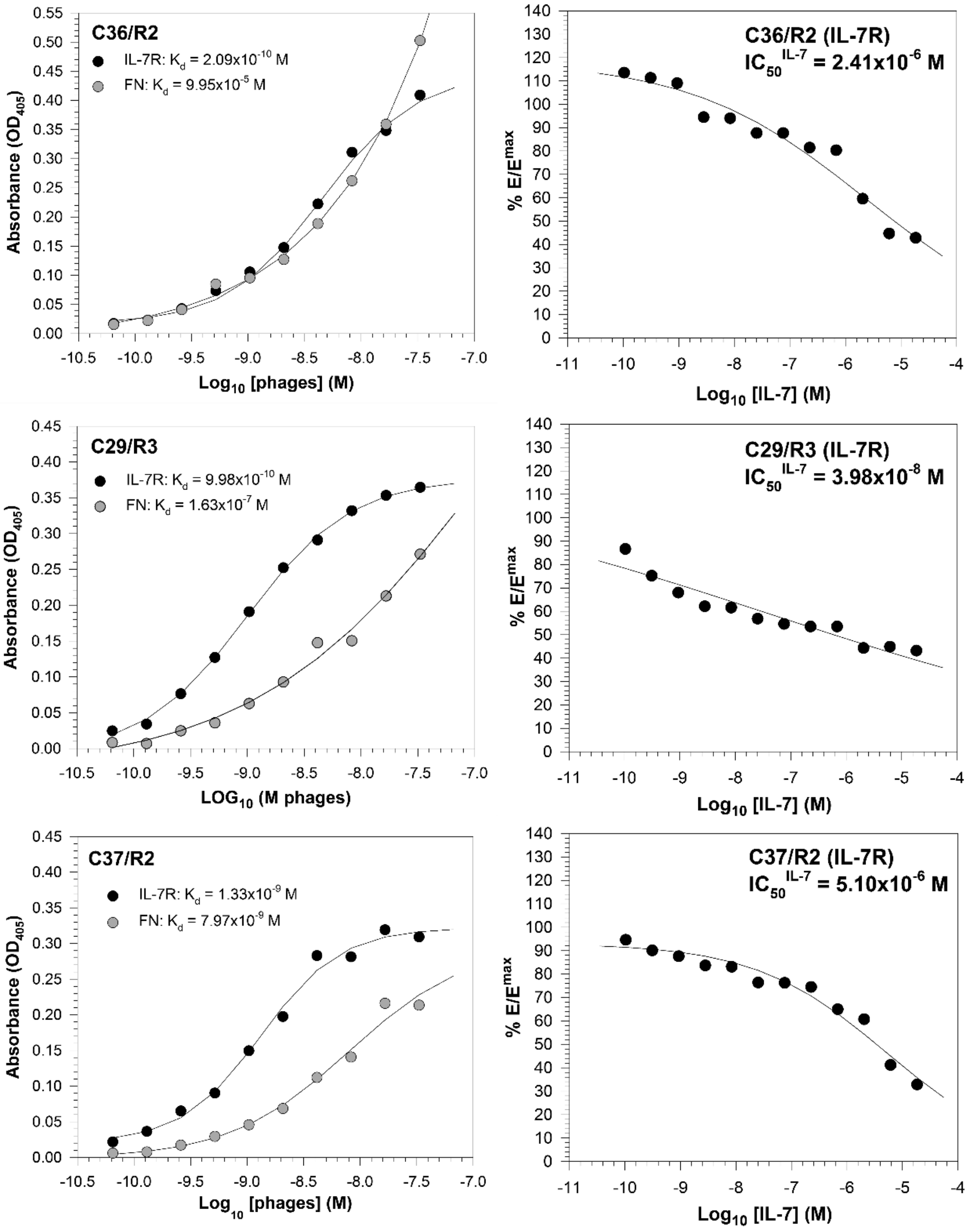

Figure S2. The affinity for IL-7Rα of the clones 36/R2, 29/R3 and 37/R2 expressed by their own K^*_d and by the IC_{50} of IL-7 as determined in competition with each clone.

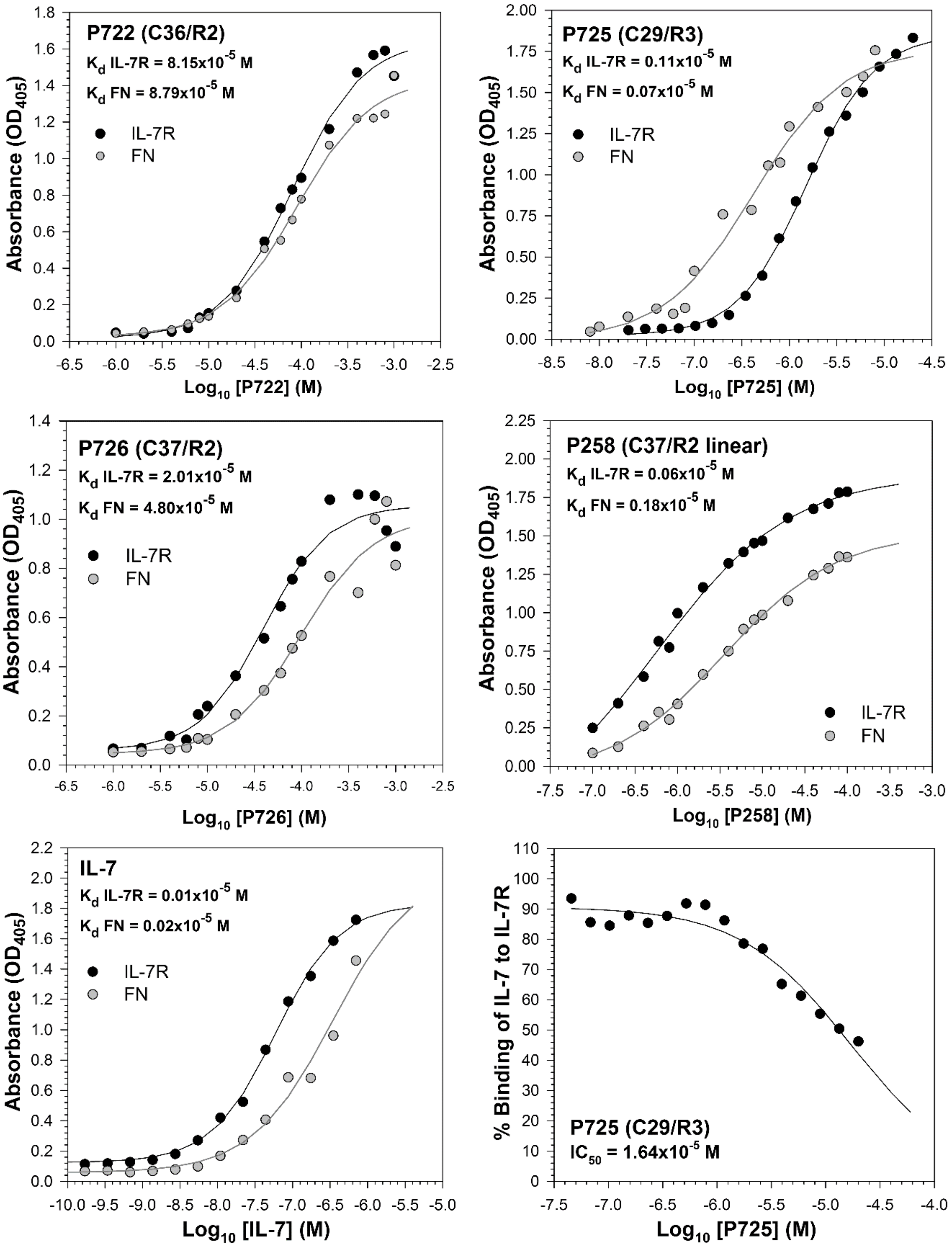

Figure S3. The affinity for IL-7Rα and FN of the candidate peptides compared to that of IL-7. The IC_{50} of P725 as determined in competition with IL-7 is also shown.

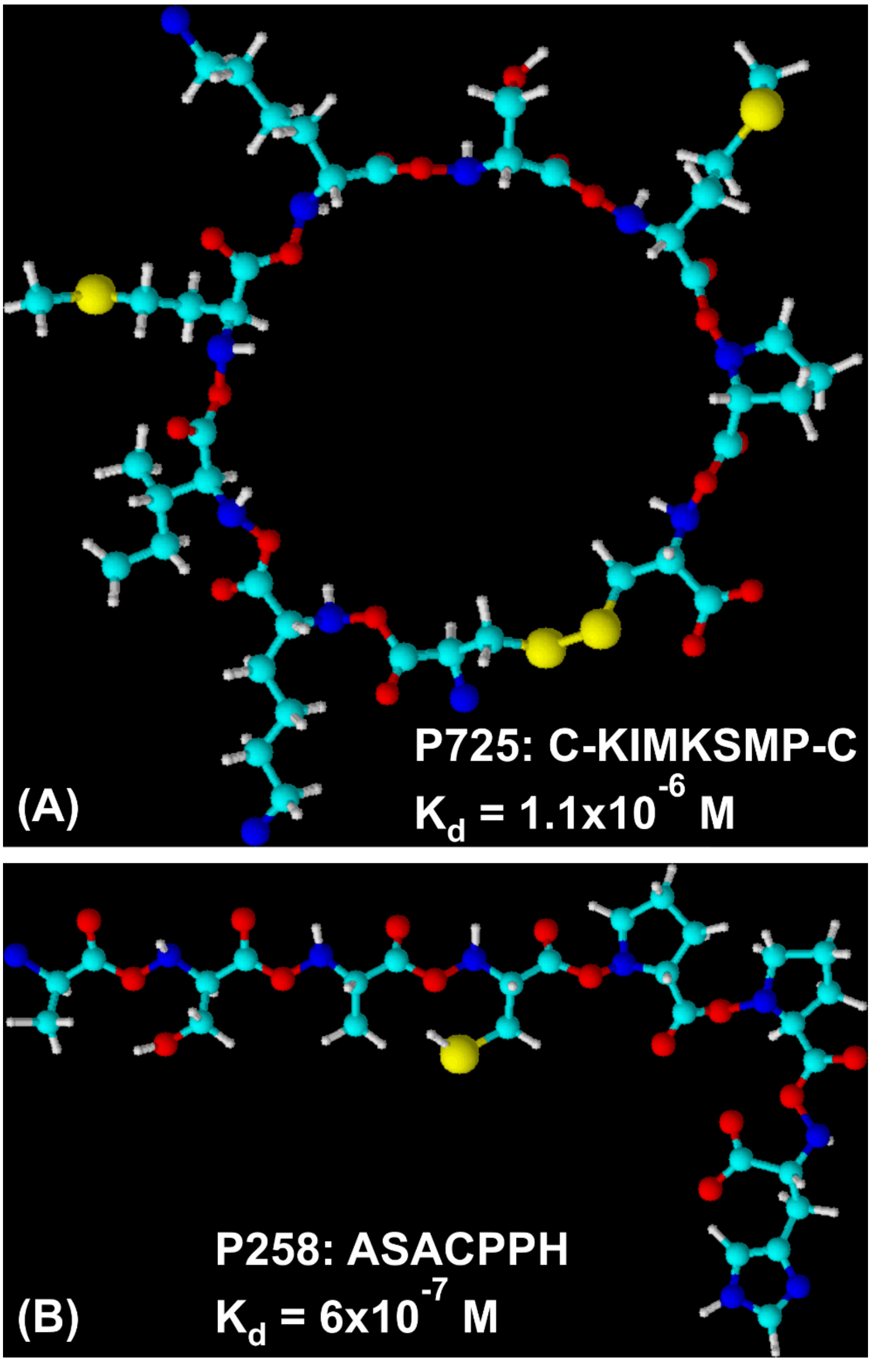

Figure S4. 3D chemical structure of peptides P725 (**A**) and P258 (**B**) (drawn with ACD/ChemSketch 2.0 software) and their K_d for IL-7Rα binding.

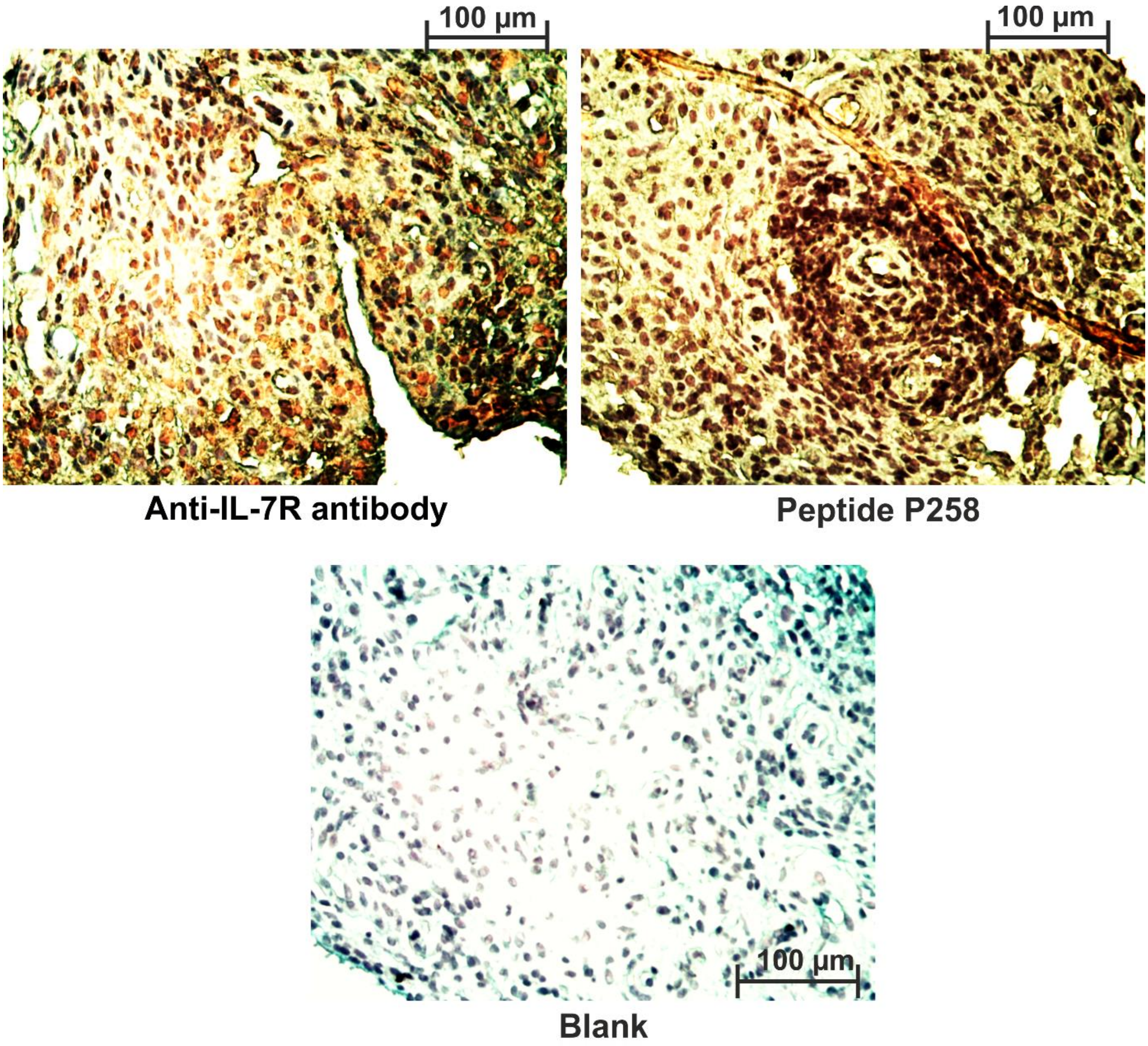

Figure S5. The expression of IL-7Rα in RA synovial tissue and the binding of biotinylated peptide P258 to the same histological tissue; each microphotograph is representative of three stained sections.

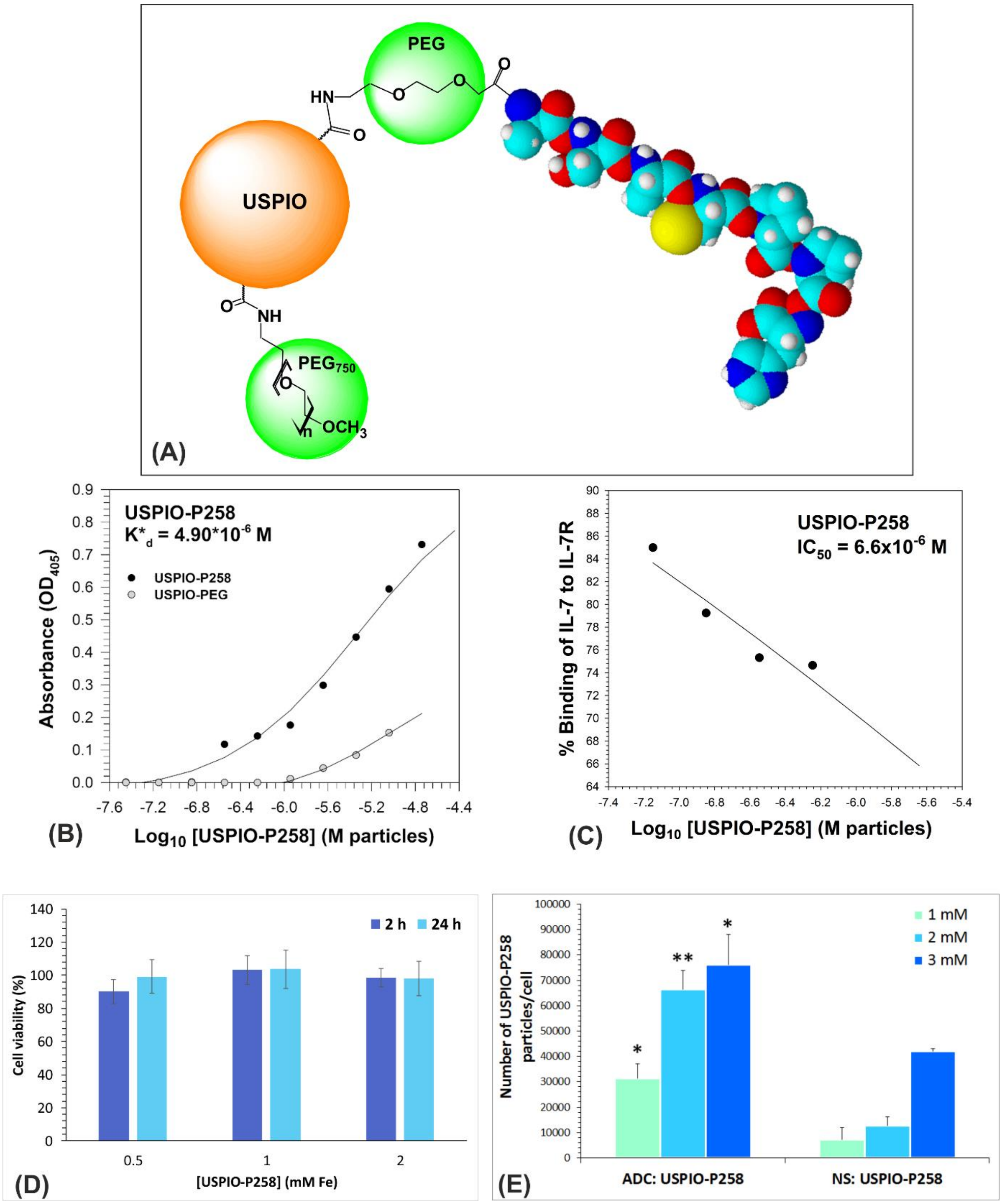

Figure S6. Affinity parameters, cytotoxicity and binding to Jurkat cells of USPIO-P258. Schematic representation of USPIO-P258 is shown in (**A**). The binding of USPIO-P258 to IL-7Rα compared to that of USPIO-PEG (**B**), its IC_{50} determined in competition with IL-7 (**C**) and its eventual cytotoxic effects evaluated on HepaRG^{TM} cell line as determined by MTT assay (**D**). The number of USPIO-P258 particles bound per ADC or NS cell is shown in (**E**); * = $p<0.05$, ** = $p<0.01$ for ADC cells vs. NS cells.

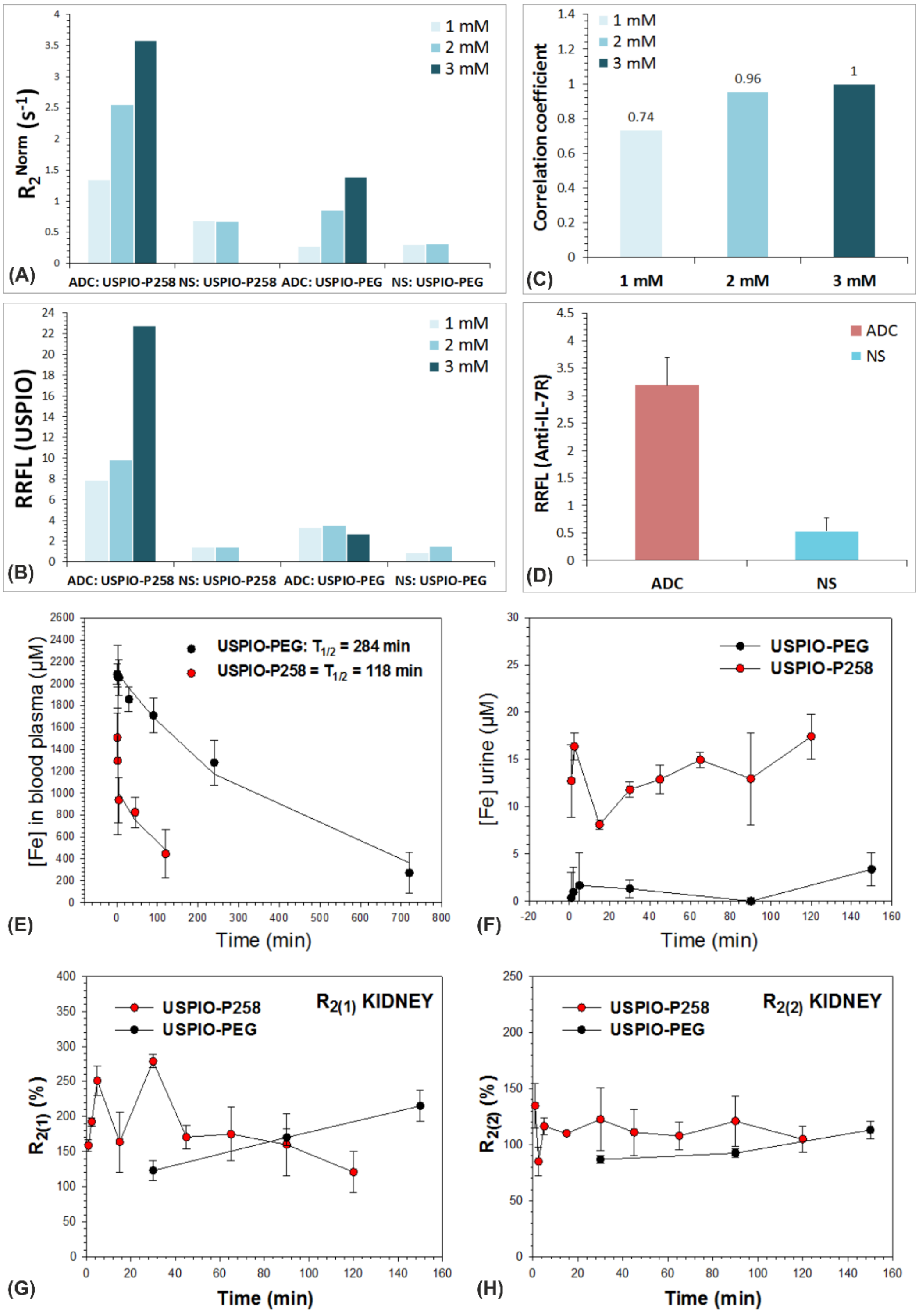

Figure S7. R_2^{Norm} (**A**) and RRFL (**B**) of ADC-stimulated (ADC) or non-stimulated (NS) Jurkat cells after incubation with USPIO-P258 or USPIO-PEG. The correlation coefficient between R_2^{Norm} and RRFL (**C**) as well as the IL-7R expression are also shown. The graphs presented in **E**-**F** illustrate the pharmacokinetics (**E**) and biodistribution of USPIO-P258 in urine (**F**) and kidney (**G** for $T_{2(1)}$ and **H** for $T_{2(2)}$) as compared to USPIO-PEG. The results for USPIO-PEG are cited from Ansciaux E et al. Contrast Media Mol Imaging. 2015; 10(3): 211-24.

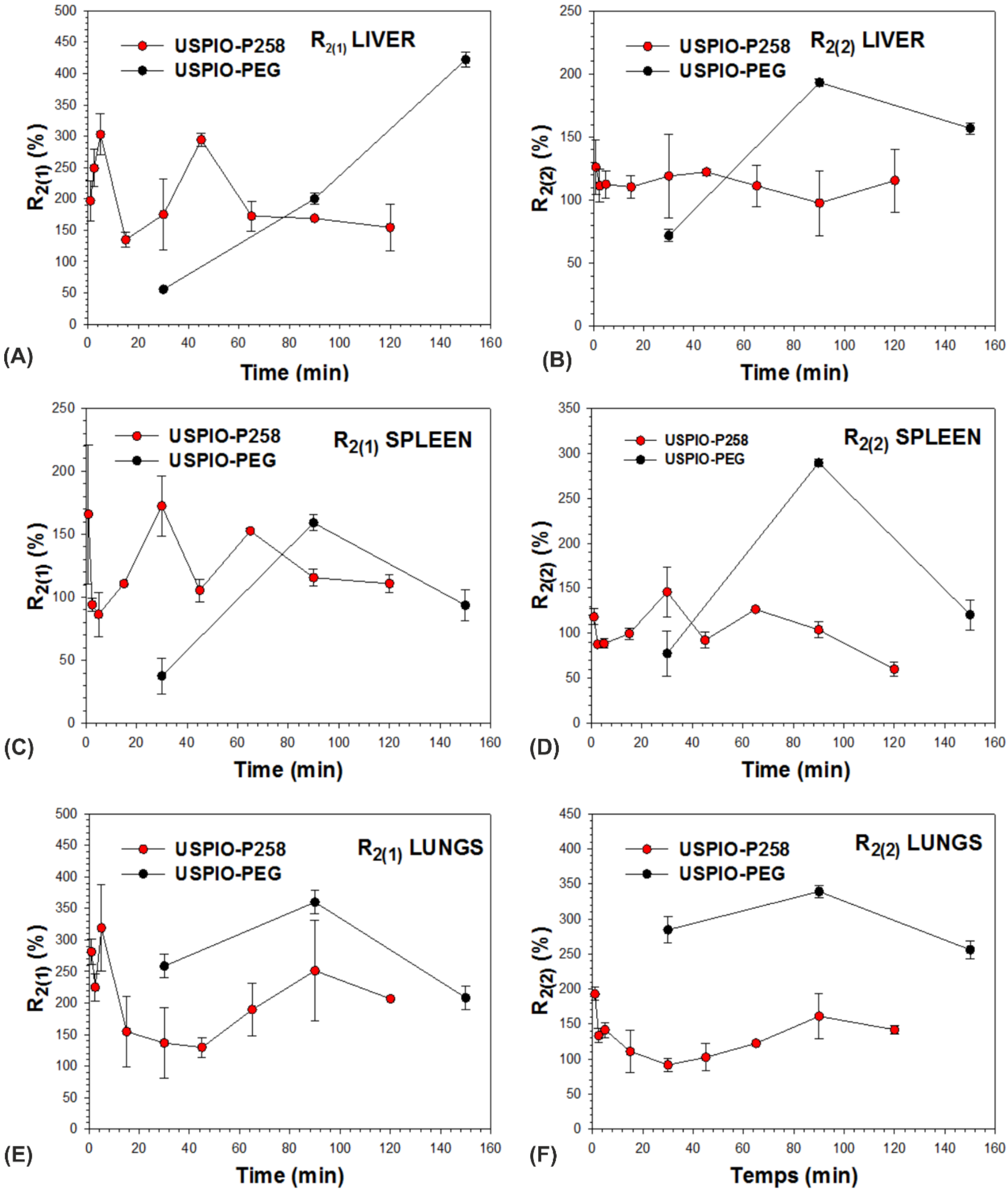

Figure S8. The biodistribution of USPIO-P258 in liver (**A** for T_{21} and **B** T_{22}), spleen (**C** and **D**) and lungs (**E** for $T_{2(1)}$ and **F** for $T_{2(2)}$) as compared to USPIO-PEG. The results for USPIO-PEG are cited from Ansciaux E et al. Contrast Media Mol Imaging. 2015; 10(3): 211-24.

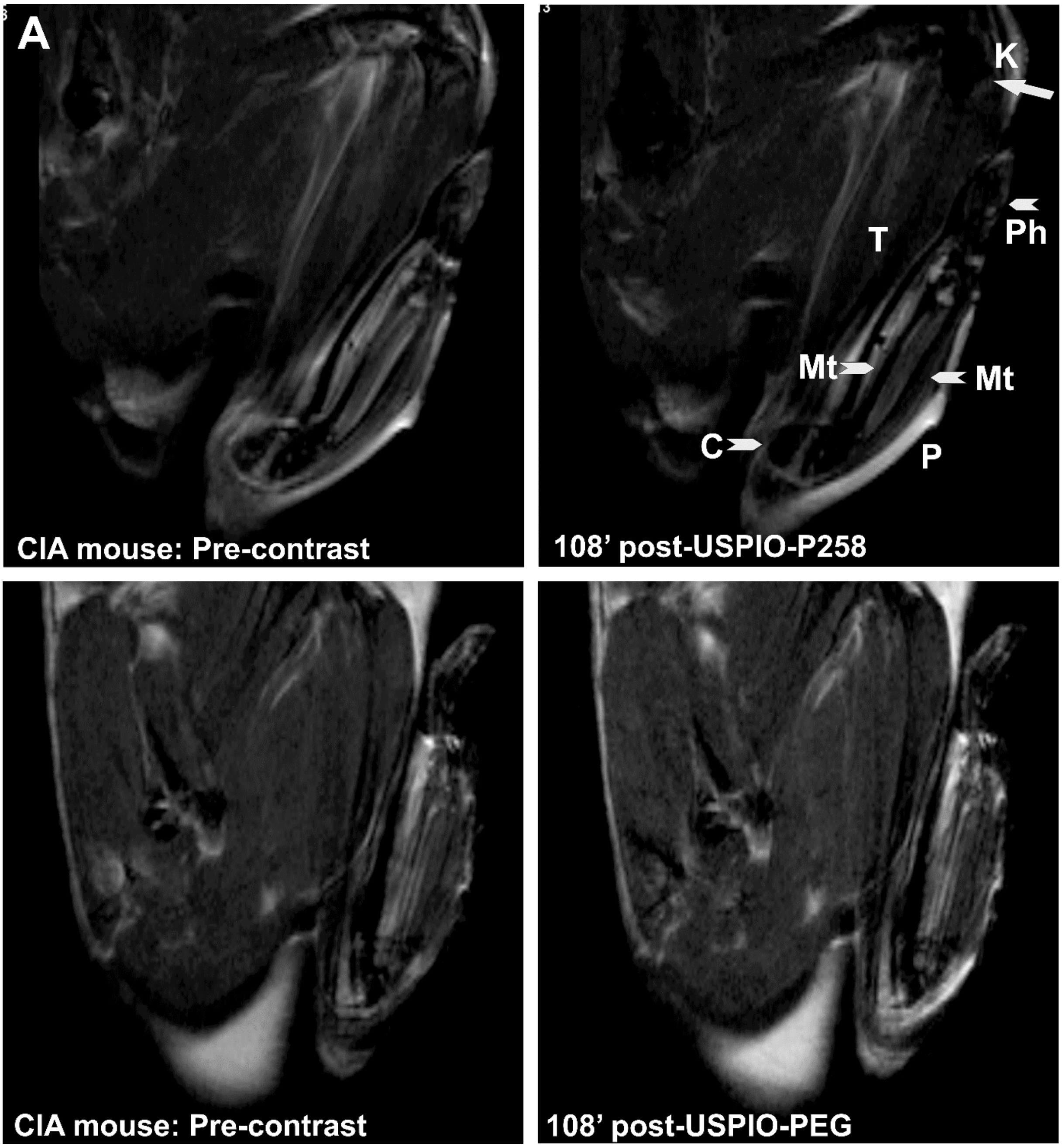
A
K
Ph
T
Mt
Mt
C
P
CIA mouse: Pre-contrast
108' post-USPIO-P258
CIA mouse: Pre-contrast
108' post-USPIO-PEG

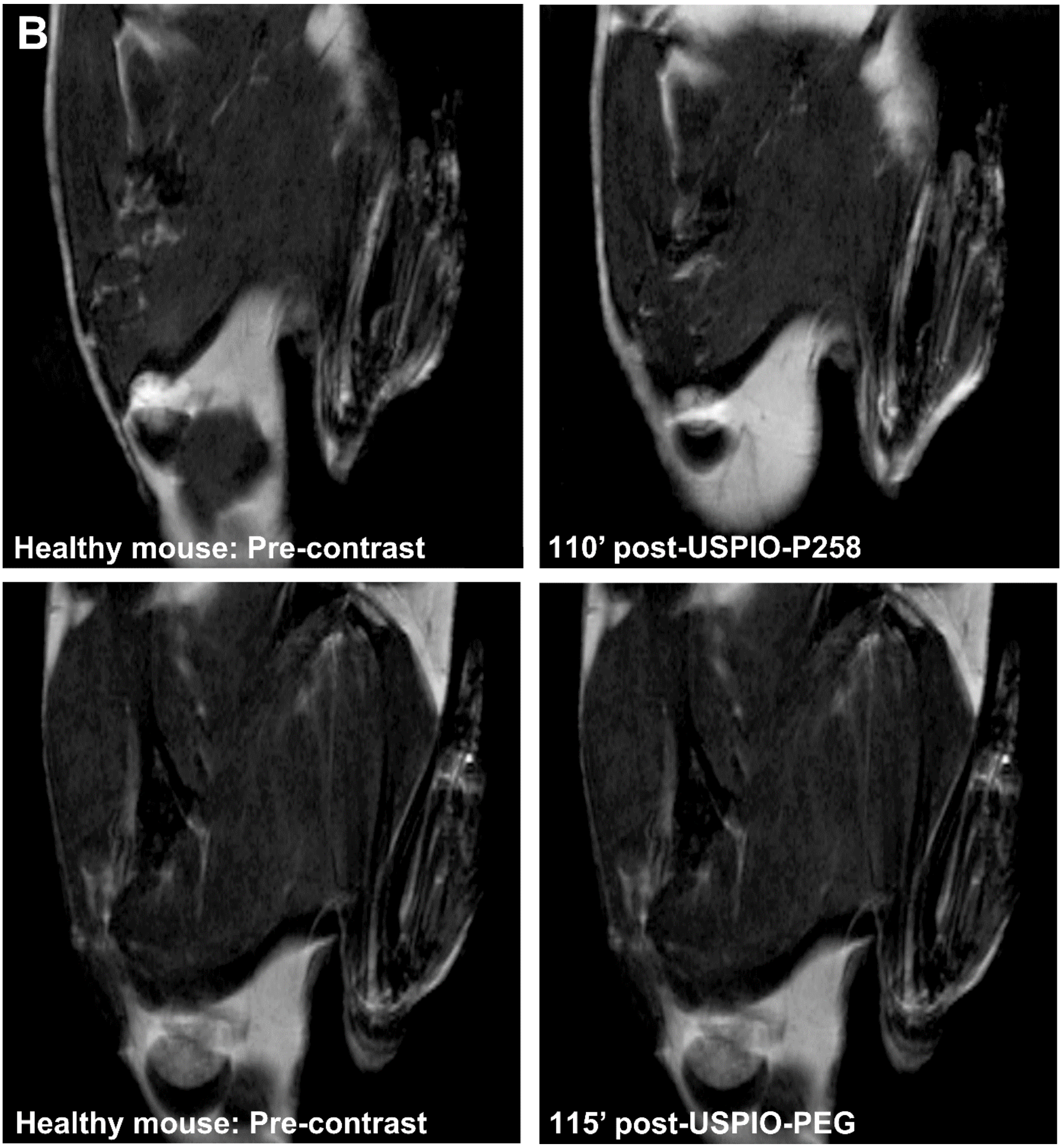

Figure S9. RARE MR images of the hind limbs of CIA (**A**) or healthy (**B**) mice injected with either USPIO-P258 or USPIO-PEG in pre-contrast and about 2 hours post-contrast. The arrowheads point to the hind limb; the arrow points to the knee. C = calcaneus; CB = coxal bones; K = knee; P = paw; Ph = phalanx; Mt = metatarsal bones; T = tibia.

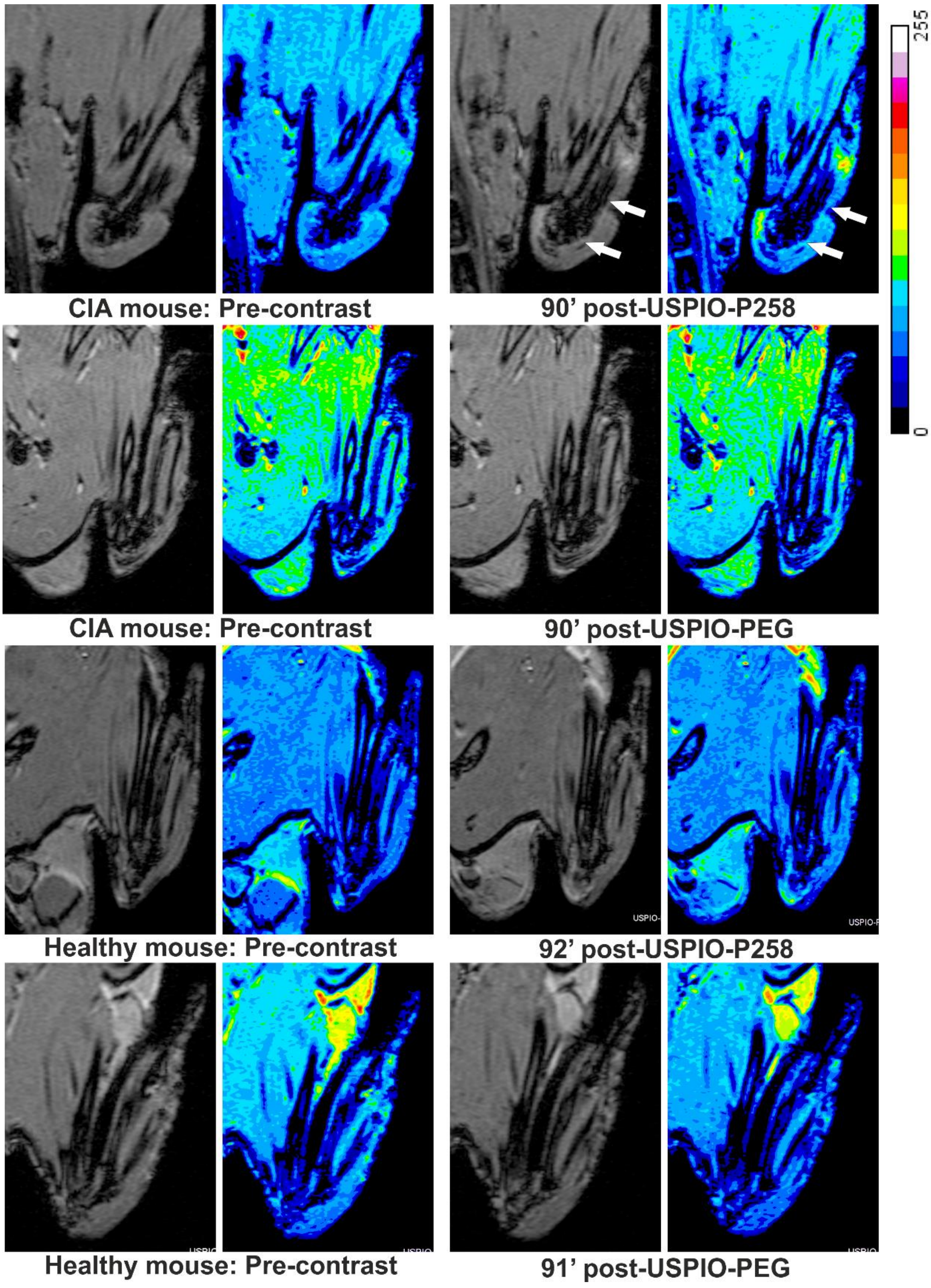

Figure S10. 3D FISP MR images of the hind limbs of CIA or healthy mice injected with either USPIO-P258 or USPIO-PEG in pre-contrast and at different times post-contrast. The color-coded signal enhancement is shown at the right side of each raw image. The arrows point to the hind limb.

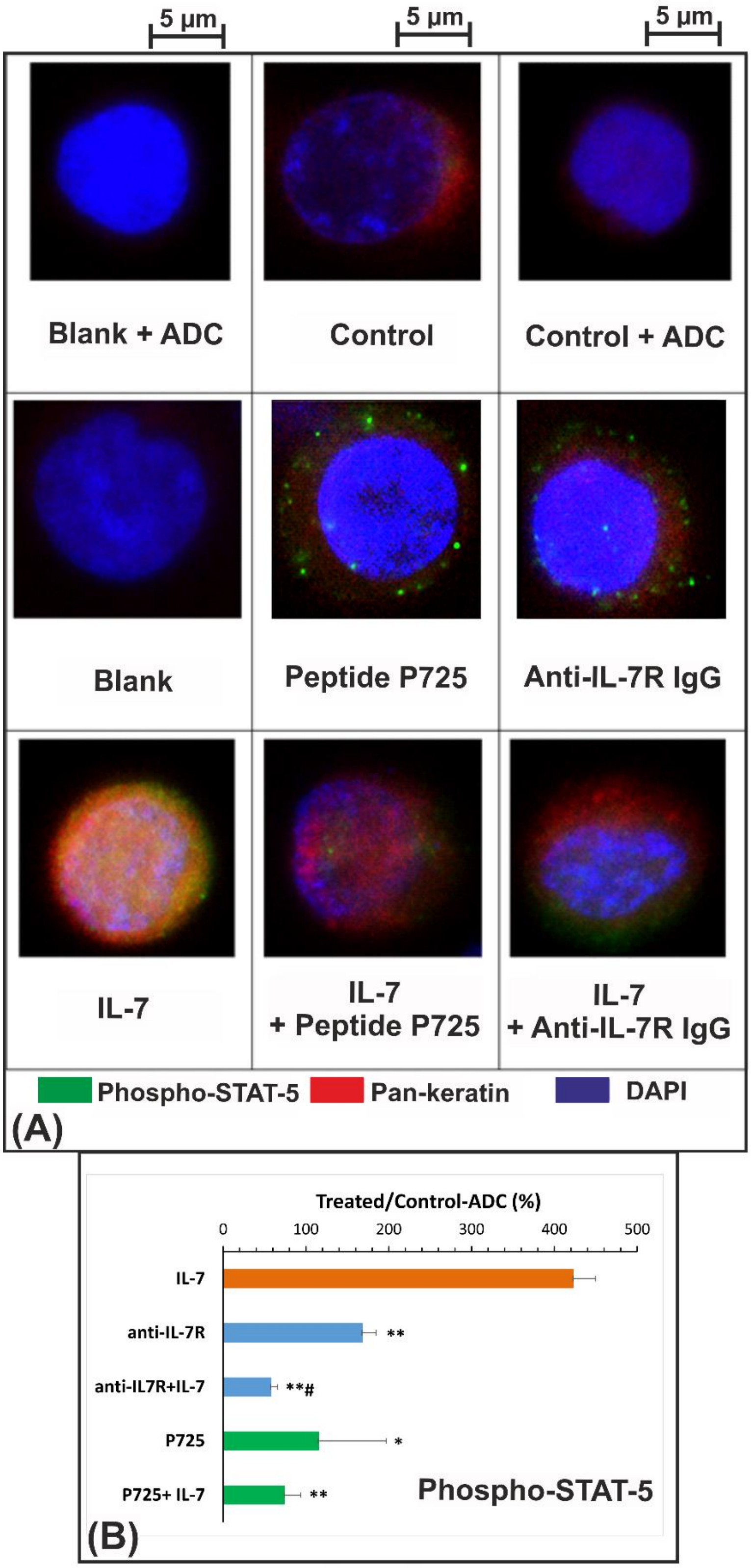

Figure S11. Immunofluorescent labeling of phospho-STAT-5 in ADC-stimulated Jurkat cells is shown in (**A**); the blank and controls are ADC-stimulated or non-stimulated Jurkat cells and incubated with (controls) or no (blank) anti-phospho-STAT-5 antibody. The semi-quantitative analysis of phospho-STAT-5 is shown in (**B**). * = $p<0.05$, ** = $p<0.01$ vs. IL-7; # = $p<0.05$ vs. anti-IL-7R.

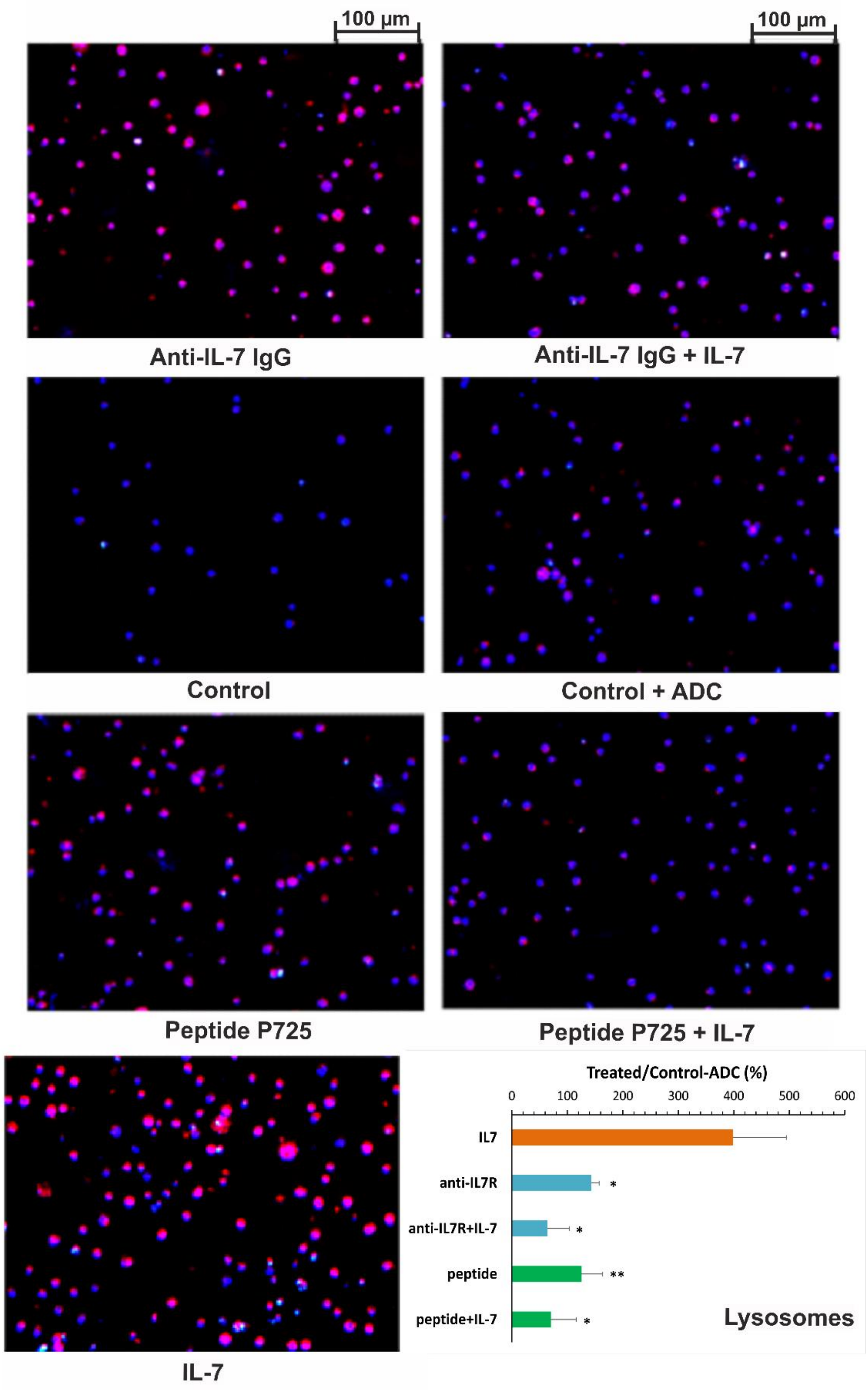

Figure S12. Lysosome detection by fluorescence on ADC-stimulated Jurkat cells and incubated with IL-7, with the competitor (anti-IL-7 antibody or peptide P725) or concomitantly with the competitor and IL-7. Controls are Jurkat cells stimulated or not with ADC. Lysosomes are stained in red with Lysotracker® Red DND-99, whereas nuclei are stained in blue with Hoechst 33342. The semi-quantitative analysis of microphotographs is shown in the histogram. * = $p<0.05$, ** = $p<0.01$ vs. IL-7.
